# Risk of dementia after bloodstream infection—a nationwide propensity score matched cohort study

**DOI:** 10.1093/ageing/afag178

**Published:** 2026-06-22

**Authors:** Jonathan Underwood, Daniel Farewell, David Gillespie, Neil A Harrison, Haroon Ahmed

**Affiliations:** Division of Infection and Immunity, Cardiff University, Cardiff CF14 4XN, UK; Department of Infectious Diseases, Cardiff and Vale University Health Board, Cardiff CF14 4XW, UK; Division of Population Medicine, Cardiff University, Cardiff CF14 4XN, UK; Centre for Trials Research, Cardiff University, Cardiff, Wales, UK; Cardiff University Brain Research Imaging Centre (CUBRIC), Cardiff University, Cardiff, Wales, UK; Division of Population Medicine, Cardiff University, Cardiff CF14 4XN, UK

**Keywords:** dementia, bloodstream infection, sepsis, propensity score matching, cohort study, older people

## Abstract

**Background:**

Dementia is a global public health threat. Current treatments have limited efficacy, making identification of modifiable risk factors of paramount importance. Observational studies link severe infections to increased dementia risk but often suffer confounding. Here, we address this by determining dementia risk following bloodstream infection (BSI) in a nationwide study.

**Methods:**

We conducted a cohort study within the Secure Anonymised Information Linkage Databank, containing anonymised population-scale electronic health record data for the population of Wales, UK. Patients with microbiologically confirmed BSI were propensity score matched 1:1 to controls (*n* = 2.5 million without dementia at baseline). We created two comparative models to estimate the true effect of BSI on dementia risk: modelling hospitalisation with sterile inflammation, uncomplicated total knee replacement (TKR) replaced BSI as the exposure; assessing residual confounding, lung cancer replaced dementia as outcome.

**Results:**

We included 26 792 people with BSI and 26 792 matched controls, all without dementia at cohort entry. BSI was associated with an increased cumulative hazard of dementia corresponding to 160 (128–182) additional cases per 1000 person-years 10 years after exposure. TKR was not associated with an increased risk of dementia. BSI was potentially associated with a small excess hazard of lung cancer [12.4 (5.4–19.5) additional cases per 1000 person-years 10 years after exposure].

**Conclusions:**

BSI was associated with incident dementia in excess of that expected by hospitalisation or residual confounding. These findings suggest that treatment of BSI and other severe infections merits further investigation as a potentially modifiable risk factor for dementia.

## Key Points

- Bloodstream infection was associated with an increased risk of dementia.
- Comparative models suggest this relationship was unlikely to be due to confounding.
- Bloodstream and other severe infections merit further investigation as potentially modifiable risk factors for dementia.

## Introduction

Dementia is a leading threat to global health affecting millions worldwide. The estimated cost to the global economy was US$1.3 trillion in 2019, with approximately half borne by informal carers [1]. While age is the most well-established risk factor for dementia, accumulating evidence suggests that certain health conditions and life events may also contribute [2]. However, established modifiable risk factors account for only ~40% of potentially preventable dementia worldwide, hence the urgent need to identify other modifiable causes. Sepsis has long been recognised as a common cause of acute cognitive impairment (known as delirium or sepsis-associated encephalopathy), but more recently has been linked to longer term cognitive impairment [3–6]. The pathophysiology is complex and multifactorial but is likely to be the result of acute and chronic systemic inflammation—both potentially modifying the trajectory of cognitive impairment in Alzheimer’s disease [7].

Population-based data from the UK has demonstrated the association between common infections and incident dementia with the risk highest for people with sepsis (*n* = 6046) [8]. Similarly, a study of intensive care unit (ICU) survivors in the USA reported severe sepsis (*n* = 3145) to be associated with an increased risk of subsequent dementia [9]. However, a larger nationwide study of ICU survivors in Sweden (*n* = 16 115 with sepsis) did not find sepsis was associated with dementia after adjusting for confounders [10]. Irrespective, these data highlight the difficulties of using observational data to investigate links between infections and dementia given the slow process of neurodegeneration, residual confounding and the potential for reverse causation. This is further complicated by bidirectional relationships between comorbidities and risk factors for dementia and infection. However, despite these difficulties determining how severe infection, where interventions are likely to be more effective and amenable to study than milder, self-limiting infection, is crucial and has been identified as a strategic goal of future dementia research by The World Health Organization (WHO) [11].

The heterogeneous aetiology of sepsis and changing international consensus definition is acknowledged as one of the biggest challenges in understanding sepsis-associated encephalopathy and its pathogenesis [12]. Bacteraemias, or bloodstream infections (BSIs), are common, life-threatening infections that cause sepsis with 30-day mortality in excess of 15%. They are associated with significant systemic inflammation with higher levels associated with greater sepsis and non-sepsis mortality [13]. Studying risk of dementia following BSI confers significant advantages over sepsis ‘per se’ given an unambiguous pathogen. However, knowledge of their long-term outcomes and risk of dementia are limited. One previous study of veterans in the USA (*n* = 2368) showed a small effect for subsequent dementia after adjustment for confounders, but with a confidence interval close to the null [HR (95% CIs): 1.22 (1.00–1.49)] [14].

We aimed to robustly determine the association between BSI and dementia in a nationwide cohort study, using additional comparative models, hypothesising that BSI would be associated with incident dementia and that this risk would differ by organism and magnitude of the systemic inflammatory response.

## Methods

### Study design and population

We performed a retrospective cohort study of adults (aged >18 years at the time of cohort entry) using the Secure Anonymised Information Linkage (SAIL) Databank dementia electronic cohort (SDEC). SDEC is a nested population-based electronic cohort (e-cohort) of people with and without diagnosed dementia within SAIL that contains anonymised, routinely collected primary care, hospital admissions, mortality and deprivation datasets for the population of Wales, UK (3 million people) [15]. Eligible participants needed to be dementia free and have SDEC data for at least a year prior to April 2010. Follow-up began on 1 April 2010 and continued until the first of: incident dementia diagnosis, death or end of SDEC e-cohort follow-up (28 February 2022).

### Data sources

SDEC methodology has previously been described in detail [15]. Briefly, dementia and its subtypes were defined using validated ICD-9, ICD-10 and Read V2 codes to identify cases from primary care, hospital admissions or mortality data (see Appendix 1.1 in Supplementary Data for further details). Blood cultures from NHS Wales institutions across primary and secondary care that grew *Escherichia coli, Klebsiella sp, Pseudomonas aeruginosa (PsA) and Staphylococcus aureus* were extracted from Public Health Wales’ data and linked to the SAIL Databank as previously described [13, 16]. More than one organism isolated from the same sample was regarded as ‘polymicrobial’ and considered separately. In the case of multiple positive blood cultures, only the first was considered for analysis.

Peak C-reactive protein (CRP), a proxy of the magnitude of the inflammatory response, was determined between the period starting 2 days before the BSI and ending 7 days after, to account for asynchronous blood draws for biochemistry and microbiological tests as previously described.

### Variables

The primary outcome was all-cause dementia occurring at least 90 days after BSI. Secondary outcomes were Alzheimer’s dementia (AD) and vascular dementia at least 90 days after BSI. Earlier diagnoses were not included to avoid coding inaccuracies, health ascertainment bias whereby admission to hospital for BSI prompted investigations for dementia, and to avoid including people with delirium, a common complication of hospitalisation with infection, misdiagnosed as dementia [8]. Exposure was a time updated variable of the first monomicrobial BSI. Polymicrobial BSI were considered separately. Potential confounders included age, sex, Charlson comorbidity index (CCI) score, Welsh Index of Multiple Deprivation (WIMD) version 2019 as quintiles mapped from lower-level super output area (LSOA) of residence (administrative authority locality), electronic frailty index (EFI) [17], alcohol dependence, obesity and ever-smoking status (see Appendix 1.2 to Appendix 1.7 in Supplementary Data for further details) at cohort entry [18]. Chosen demographic, socioeconomic, comorbidity and lifestyle covariates could all potentially confound the relationship between BSI and dementia and were available in a standardised format at the population level.

### Statistical analyses

As the population with BSI was significantly older and more-comorbid than the population without BSI, we used 1:1 propensity score matching to create balanced exposed and unexposed groups rather than inverse probability weighting (IPW) given the risks of extreme weights. This was accomplished by calculating a propensity score from a binary logistic regression model where the exposure (BSI) was conditioned on all potential confounders (listed above) as well as cohort start year strata; hence, there was no imputation for missing data. Covariate balance was assessed using standardised mean differences. Incident dementia was the primary outcome. Death or alive at follow-up were right-censored. We originally used adjusted Cox regression with incident dementia as the dependent variable and time-updated BSI status as the independent variable to estimate the association between BSI and incident dementia. However, proportional hazards assumptions, assessed by plotting the scaled Schoenfeld residuals against time, were not met (see Appendix 2.12 in Supplementary Data for further details) in keeping with a similar study using this methodology [8]. As such we used additive regression with the Aalen model, which does not assume proportional hazards, to determine the risk of dementia over time. The model reports cumulative coefficients that represent the cumulative excess hazard attributable to BSI up to a certain time. Deaths were treated as censoring, and the analysis, therefore, estimates dementia-specific cumulative excess hazard among survivors. Cumulative excess hazards were expressed as additional cases per 1000 person-years after 1, 5 and 10 years of follow-up. To avoid matching model misspecification, potential confounders were also included in the primary analysis using the Aalen model—so called ‘double robustness’. Because of matching, we computed cluster-robust standard errors using the matched pair identifier. For transparency, unadjusted analyses are also reported. Uniform confidence bands were calculated using bootstrapping with 1000 simulations.

Further exploratory analyses using similar adjusted Aalen models were undertaken to assess dementia risk after BSI stratified by the magnitude of inflammatory response and BSI organism (divided into Gram-negative and Gram-positive organisms and removing polymicrobial BSI).

### Pre-planned sensitivity analyses and comparative models

To account for reverse causation, mis-coding and ‘unmasking’ effects of BSI and hospitalisation on the diagnosis of dementia, a sensitivity analysis where incident dementia occurring at least 365 days after BSI was used as the outcome in the additive regression models. Hospitalisation with BSI is a complex event involving many exposures that may increase the risk of illness and dementia (e.g. systemic inflammation). To estimate, the effect of hospitalisation with ‘sterile inflammation’, we used the same initial dataset and methodology to determine the risk of dementia following admission for total knee replacement (TKR). We excluded patients who were re-admitted 90 days after TKR or who developed infective complications, classifying the rest as ‘uncomplicated’ TKR (see Appendix 1.7 in Supplementary Data for further details). This was used as the exposure instead of BSI, again as a time-updated covariate in adjusted Aalen models. Unmeasured confounders are a problem with observational research. As such, we used the same data set and methodology to assess the relationship between BSI and lung cancer as a ‘negative control’ outcome. This outcome was chosen as there was no biologically plausible reason why BSI should be associated with subsequent lung cancer (ICD-10 code C34). However, it was conceivable that if confounding was not suitably addressed by matching and model adjustment, then residual confounding may result in a spurious association between BSI and lung cancer. Furthermore, this method may also help explore health ascertainment bias, whereby hospitalisation for BSI results in frequent medical review and investigations that may diagnose previously asymptomatic pathology like dementia or lung cancer. We triangulated findings from these complementary analyses in our interpretation.

All statistical analyses were conducted using R v4.3.3 using survival and timereg v2.0.7 packages [19].

## Results

We included 26 792 people who developed BSI with no diagnosis of dementia at cohort entry [median age 67 years, 14 392 (54%) males, 16 108 (60%) *E. coli,*  Table 1]. We successfully matched 26 792 controls from a population of 2 450 471 people without dementia from SDEC (standardised mean distance 0.000, Appendix 2.1 in Supplementary Data). A total of 4688 new cases of dementia were diagnosed during follow-up, 1520 and 1512 classified as Alzheimer’s or vascular dementia, respectively.

**Table 1:** Demographics.

	Bloodstream infection		
Variable	No BSI *N* = 26 792^a^	BSI *N* = 26 792^a^	Standardised mean difference
**Age (years)**	67 (56–77)	67 (55–76)	−0.049
**Sex**			−0.007
*Male*	14 215 (53)	14 392 (54)	–
* Female*	12 577 (47)	12 400 (46)	–
**Charlson Index**			0.12
* Up to 20*	26 555 (99)	26 453 (99)	–
* >20*	240 (0.9)	342 (1.3)	–
**Welsh index of multiple deprivation**			
* 5 (least deprived)*	4664 (18)	4651 (17)	−0.001
* 4*	4951 (18)	4893 (18)	−0.002
* 3*	5273 (20)	5251 (20)	−0.001
* 2*	6002 (22)	55 958 (22)	−0.002
* 1 (most deprived)*	5902 (23)	6039 (23)	0.005
**Frailty rating**			
* Fit*	14 418 (54)	14 219 (53)	−0.010
* Mild*	9139 (34)	8659 (32)	−0.018
* Moderate*	2749 (10)	3306 (12)	0.021
* Severe*	486 (1.8)	698 (2.6)	0.008
**Smoking**			−0.008
* Non-smoker*	9358 (36)	9574 (36)	–
* Smoker*	17 434 (64)	17 218 (64)	–
**Alcohol excess**			0.007
* Alcohol excess*	1518 (5.7)	1709 (6.3)	–
* No alcohol excess*	25 274 (94)	25 083 (94)	–
**Obesity**			0.014
* Not obese*	23 250 (87)	22 887 (85)	–
* Obese*	3542 (13)	3905 (15)	–
**BSI organism**			NA
* E. coli*	–	16 108 (60)	–
* Klebsiella*	–	2952 (11)	–
* MRSA*	–	734 (2.7)	–
* MSSA*	–	5475 (20)	–
* Polymicrobial*	–	532 (2.0)	–
* PsA*	–	991 (3.7)	–
**Duration of follow-up (years)**	10.5 (4.9–11.9)	9.5 (5.5–11.9)	NA
**Cohort start year**			
* 2010–2013*	26 090 (97)	25 869 (97)	−0.008
* 2014–2017*	598 (2.3)	752 (2.8)	0.006
* 2018–2022*	104 (0.4)	171 (0.6)	0.003
**Outcome**			NA
* Alive, no dementia*	15 425 (58)	11 373 (42)	–
* Developed dementia*	2934 (11)	1754 (6.5)	–
* Died*	8433 (31)	13 665 (51)	–

^a^Median (IQR); *n* (%)

### BSI is associated with incident dementia

**Figure 1:**
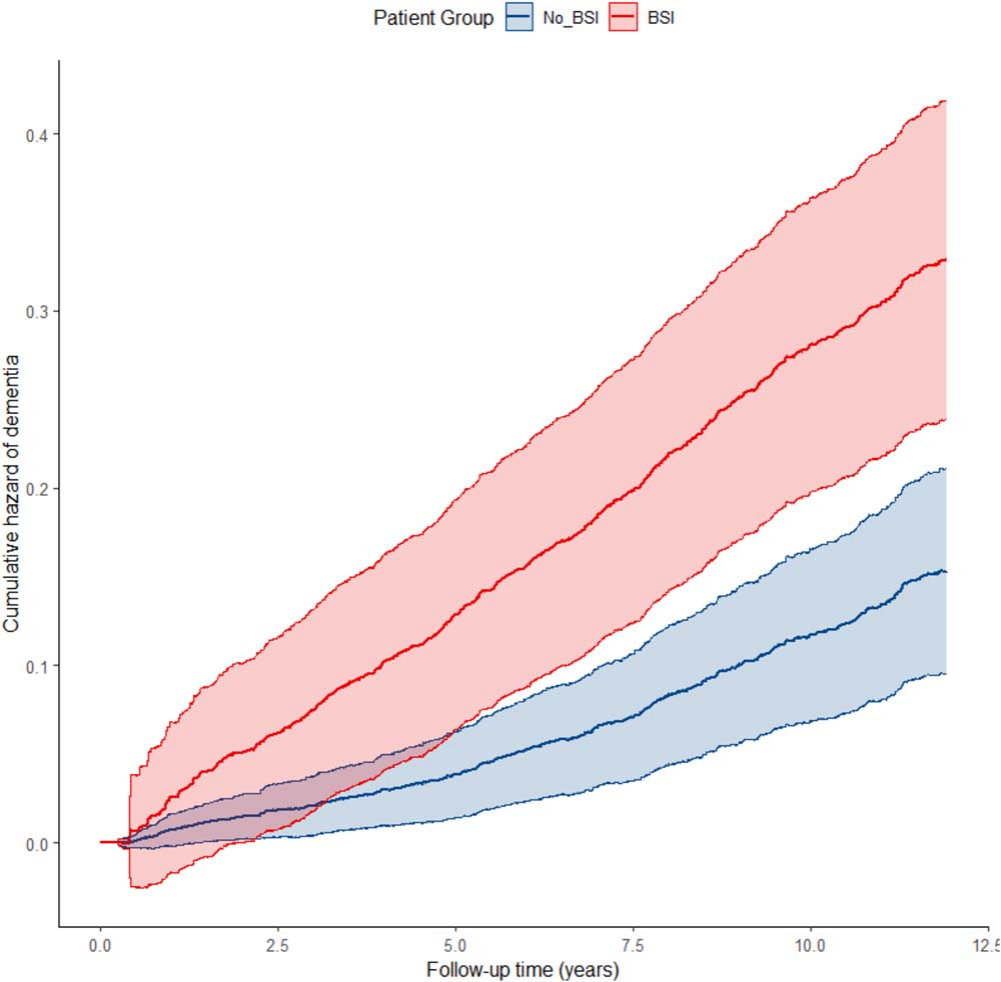
BSI is associated with an increased risk of dementia. Cumulative hazard plot of baseline dementia risk and excess associated with BSI—from adjusted additive regression mode of propensity score matched cohort. Plotted with uniform 95% confidence intervals, whereby the confidence level applies simultaneously across the entire range of the curve, not just at individual time points.

BSI was associated with an increase in cumulative hazard of dementia (Supremum-test 15.2, *P* < .001), and this effect was time varying (Kolmogorov–Smirnov test 0.024, *P* = .002, Figure 1, Table 2, Appendix 2.2). BSI was associated with both incident Alzheimer’s (Supremum-test 14.9, *P* < .001) and vascular dementia (Supremum-test 8.2, *P* < .001); however, the cumulative excess hazard was higher for vascular dementia (Table 2, Appendices 2.3 and 2.4 in Supplementary Data).

**Table 2:** Cumulative excess hazard of dementia after BSI.

		Unadjusted	Adjusted	
	Time (years)	Cumulative excess hazard (95% CI)	Cumulative excess hazard (95% CI)	Excess dementia (per 1000 patient years)
	1	0.020 (0.006–0.034)	0.018 (0.005–0.032)	18 (4.7–32)
**All cause dementia**	5	0.094 (0.074–0.114)	0.087 (0.067–0.107)	87 (67–107)
	10	0.166 (0.144–0.189)	0.160 (0.138–0.182)	160 (128–182)
	1	0.002 (−0.003–0.006)	0.002 (−0.003–0.006)	1.5 (−0.3–6.2)
**Alzheimer’s dementia**	5	0.012 (0.005–0.019)	0.011 (0.004–0.018)	10.7 (3.8–17.6)
	10	0.029 (0.021–0.037)	0.029 (0.021–0.037)	28.9 (20.5–37.3)
				
	1	0.004 (−0.004–0.012)	0.003 (−0.005–0.011)	3.4 (−0.5–11.0)
**Vascular dementia**	5	0.023 (0.017–0.038)	0.025 (0.014–0.035)	24.8 (14.2–35.0)
	10	0.050 (0.038–0.062)	0.048 (0.036–0.059)	47.5 (35.7–59.0)

In a pre-specified sensitivity analysis where dementia was diagnosed at least 365 days after BSI, the association between BSI and incident dementia remained (Supremum-test 12.0, *P* < .001). However, the magnitude of the association was reduced [126 (104–150) excess cases per 1000 person-years, 10 years after BSI].

### Using TKR as the exposure

**Figure 2:**
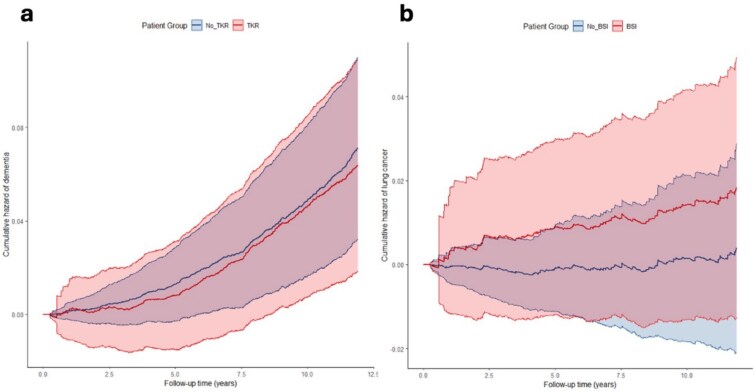
Comparative models showing risk of dementia following uncomplicated TKR and risk of lung cancer following bloodstream infection. Cumulative hazard plots of baseline dementia risk and excess associated with TKR (a) and baseline lung cancer risk and excess associated with BSI (b)—from adjusted additive regression models on separate propensity matched cohorts. Plotted with uniform 95% confidence intervals, whereby the confidence level applies simultaneously across the entire range of the curve, not just at individual time points.

A total of 21 882 people with TKR [median age 64, 9365 (43%) male, see Appendix 2.13 in Supplementary Data for further demographics] were identified and successfully matched to 21 882 controls (standardised mean difference 0.000, Appendix 2.5 in Supplementary Data). In unadjusted models, TKR was associated with a small excess cumulative hazard of dementia. However, this was no longer present after adjustment for potential confounders, and TKR was associated with a possible small protective effect (Supremum-test 5.17, *P* < .001, Figure 2a, Table 3, Appendix 2.6 in Supplementary Data).

**Table 3:** Comparative models: risk of dementia following uncomplicated TKR and risk of lung cancer following BSI.

		Dementia		
		Unadjusted	Adjusted	
	Time (years)	Cumulative excess hazard (95% CI)	Cumulative excess hazard (95% CI)	Excess dementia (per 1000 patient years)
	1	0.001 (−0.003–0.004)	−0.001 (−0.005–0.002)	−1.3 (−4.5–18)
**Uncomplicated TKR**	5	0.007 (0.001–0.012)	−0.004 (−0.009–0.001)	−4.2 (−9.4–1.0)
	10	0.023 (0.015–0.030)	−0.003 (−0.010–0.004)	−2.8 (−9.8–4.3)
		Lung cancer		
		Unadjusted	Adjusted	
	Time (years)	Cumulative excess hazard (95% CI)	Cumulative excess hazard (95% CI)	Excess lung cancer (per 1000 patient years)
	1	0.003 (−0.002–0.008)	0.003 (−0.002–0.008)	3.0 (−1.7–7.7)
**Bloodstream infection**	5	0.010 (0.003–0.016)	0.010 (0.035–0.016)	9.9 (3.5–16.3)
	10	0.012 (0.005–0.019)	0.012 (0.005–0.020)	12.4 (5.4–19.5)

### Using lung cancer as the outcome

A total of 29 054 people had BSI before any diagnosis with lung cancer [median age 68 years, 15 564 (54%) male, see Appendix 2.14 in Supplementary Data for further demographic details]. They were successfully matched to 29 054 controls (standardised mean difference 0.000, Appendix 2.7 in Supplementary Data). BSI was associated with a small excess hazard of lung cancer (Supremum-test 5.65, *P* < .001, Figure 2b, Table 3, Appendix 2.8 in Supplementary Data) corresponding to approximately 12.4 (5.4–19.5) additional cases of lung cancer 10 years after BSI.

In pre-specified exploratory analyses, there was no difference in dementia risk between Gram-positive and Gram-negative organisms (Supremum-test 2.51, *P* = .27, Appendices 2.9, 2.15 and 2.16 in Supplementary Data). However, peak CRP after BSI was negatively associated with risk of dementia (Supremum-test 6.56, *P* < .001, Appendices 2.10, 2.11, 2.17 and 2.18 in Supplementary Data).

## Discussion

In this nationwide, population-based study of 2.5 million people, we found that BSI was associated with incident dementia. This relationship persisted even after extending the time from BSI to dementia to over a year, suggesting that this relationship is robust to reverse causation and that BSI contributes to neurodegeneration.

Our main finding is consistent with the one previous study suggesting BSI was associated with subsequent dementia and other data reporting a similar magnitude association with sepsis [8, 9, 14]. In contrast, a recent nationwide Swedish study of ICU survivors did not show an association between sepsis and subsequent dementia [10]. Ahlström and colleagues studied survivors of ICU and compared people with a sepsis diagnosis code with people who did not. People with traumatic brain injuries or other confounding neurological conditions that could have conceivably led to their index ICU admission were not excluded. Although, survivors of sepsis had higher rates of incipient dementia, sepsis did not remain independently associated with dementia after adjusting for covariates—many of which would be associated with sepsis itself (e.g. severity of illness score, renal replacement therapy invasive ventilation), which may tend findings towards the null.

Neurodegeneration manifesting in dementia typically occurs over many years before symptoms manifest. Given this, how are our findings plausibly explained pathophysiologically? It is likely that BSI provides a significant inflammatory ‘stress-test’. Vulnerable individuals develop delirium, unmasking their previously asymptomatic dementia and reducing the time to diagnosis. However, risk of dementia following BSI remained even for more distant diagnoses, suggesting there may be causal link. Recent data has demonstrated that delirium is associated with a three-fold increased risk of subsequent dementia with more frequent episodes further increasing risk [20]. Animal models suggest that amyloid and tau pathology can develop swiftly after an inflammatory result simulating Gram-negative infection [21, 22]. These changes are mediated by exaggerated inflammatory responses to microglia ‘primed’ by Alzheimer’s like pathology. The hippocampus seems particularly vulnerable to both age-related changes in BBB function and sepsis [23–25]. This intersection may be particularly pertinent in older people with severe infections and may explain some of the pathophysiology of infection-associated encephalopathy and subsequent neurodegeneration. Together, it is likely that pre-existing brain injury alters the host response to systemic infection, making the brain more susceptible to inflammatory stimuli, further contributing to neurodegeneration in a vicious cycle.

Additionally, the procoagulant effects of sepsis are well documented, as well as associations between common infections and incident cerebrovascular disease [26]. For example, influenza-like illness is associated with a short-term increased risk of ischaemic stroke and influenza vaccine with a reduction in stroke risk [27, 28]. Furthermore, we have previously reported the increased short-term risk of stroke following BSI [29]. Consistent with this hypothesis, we found the relationship between BSI and vascular dementia was stronger than with AD. These results are consistent with Muzambi *et al.,* which used comparable methodology [8]. Confirmation of AD pathology with CSF biomarkers, amyloid/tau PET imaging or post-mortem examination was rare in the UK during the study period so these differential relationships should be interpreted with caution. Furthermore, in reality mixed dementia predominates and there is emerging evidence showing neurovascular changes predate AD and clinical dementia suggesting that AD and vascular disease are intertwined [30, 31].

Differences in structural components of Gram-positive and Gram-negative cell walls affect pathogen-associated molecular patterns (PAMPs) recognised by the host’s immune system. Greater microglial activation and apoptosis has been reported in Toll-like receptor (TLR) 4 compared with TLR2 stimulation in murine models [32, 33]. However, in adjusted analyses, we did not see any difference in subsequent dementia risk. This may be explained by differences in upstream PAMP signals being dwarfed by the significant inflammation and hospitalisation (common in all BSI) in terms of subsequent dementia risk. The finding that lower peak inflammation is associated with increased dementia risk is harder to explain. We hypothesised that greater inflammation would be associated with increased risk of dementia due to a greater central nervous system (CNS) inflammatory insult. This finding may be explained by a more robust initial inflammatory response having less adverse homeostatic effects on the long-term balance between pro- and anti-inflammatory pathways, which may be important in long-term outcomes like dementia. However, we do not have data to test this hypothesis. Alternatively, this may represent residual confounding or attenuated inflammatory responses in people more at risk of dementia as patients with higher peak CRP were younger and less comorbid. However, these effects are complicated by the association between peak CRP and mortality after BSI, thereby reducing subsequent ascertainment of dementia. As such, these exploratory analyses should be interpreted cautiously. Confirmation in other cohorts and further mechanistic studies are warranted as this may provide useful therapeutic insights.

Our study has several limitations. The primary outcome relied on a coded diagnosis of dementia, which likely significantly underestimates the true burden of disease. Similarly, we do not have data on how dementia was diagnosed and use of more advanced imaging to diagnose dementia subtypes. As such, our secondary analyses, looking at dementia subtypes, should be interpreted with caution. As this is an observational study, definitively determining causation is not possible. Despite matching and double adjustment, our lung cancer analysis suggests a degree of residual confounding or health ascertainment bias that may inflate the association between BSI and dementia. However, the magnitude of this effect was small, suggesting the observed association between BSI and dementia is unlikely to be fully explained by these confounding factors. Reverse causality is possible given the significant asymptomatic neurodegeneration that must occur prior to diagnosis of dementia. Although our exclusion of dementia diagnosed up to 90 days after BSI, sensitivity analysis looking at dementia diagnosed more than a year after BSI, as well as accounting for frailty and not just comorbidities, mitigates this somewhat. A key limitation is the high mortality after bloodstream infection, which may influence how the association with dementia is interpreted. In our analyses, death was treated as a censoring event, so the results estimate the excess rate of dementia among people who survived and remained under follow-up, rather than the overall observed probability of developing dementia in the whole cohort. Because more patients with BSI died during follow-up, the absolute cumulative incidence of dementia in the full exposed group may differ from the survivor-based estimates reported here. Other limitations include limited information on specific diagnoses (specifically CNS infections). However, given our choice of BSI organisms, primary CNS infection would be very unlikely. We have no data on treatment, which may be an important modifier of subsequent dementia risk. Similarly, we lacked physiological and organ function data to confirm the presence of sepsis. However, it is likely a significant proportion of our cohort would meet sepsis 3 criteria. Furthermore, we lacked data on illness severity and length of hospital stay. These are downstream consequences of BSI but also contain information about pre-existing vulnerability. Adjusting for them could have removed part of the association of interest, although some residual confounding by baseline frailty or vulnerability may remain. Our lung cancer analysis quantifies this to allow comparison with the results of our primary analyses. Further mechanistic research is needed to determine whether the pathogen, infection syndrome, host-response or some combination thereof is most important for determining future outcomes. Immunomodulatory therapy is seldom used alongside antibiotics to treat BSI due to multiple failed trials in sepsis and lack of BSI-specific data. This presents an opportunity for new treatment paradigms to reduce the long-term sequelae of significant infections. Additionally, further research into infection prevention strategies like vaccines should also be explored given their potential to reduce dementia risk [34].

## Conclusion

In this nationwide cohort study, we found BSI was associated with incident dementia. Further understanding of infection-associated encephalopathy as a modifiable risk factor for dementia is imperative given an increasingly older and comorbid population.

## Supplementary Material

aa-26-0487-File002_afag178

## Research Data Transparency and Availability

The data used in this study are available in the SAIL Databank at Swansea University, Swansea, UK, but as restrictions apply, they are not publicly available. All proposals to use SAIL data are subject to review by an independent Information Governance Review Panel (IGRP). The SAIL Databank IGRP approved the study, project approval numbers 0923 and 0972. Before any data can be accessed, approval must be given by the IGRP. The IGRP carefully considers each project to ensure the proper and appropriate use of SAIL data. When access has been granted, it is gained through a privacy-protecting trusted research environment (TRE) and remote access system referred to as the SAIL Gateway. SAIL has established an application process to be followed by anyone who would like to access data via SAIL at https://www.saildatabank.com/application-process.
