# Supplementary material for: Risk of dementia after bloodstream infection—a nationwide propensity score matched cohort study
Source: Age Ageing. 2026 Jun 22;55(6):afag178. doi: 10.1093/ageing/afag178 (PMC13284705; doi:10.1093/ageing/afag178)
Supplement: aa-26-0487-File002_afag178 [file aa-26-0487-file002_afag178.docx]

Risk of dementia after bloodstream infection – a nationwide propensity score matched cohort study

**Contents**

[Appendix 1. Supplementary methods 3](#_Toc230786730)

[Appendix 1.1. Dementia codes 3](#_Toc230786731)

[Appendix 1.1.1. Code selection from SDEC 3](#_Toc230786732)

[Appendix 1.1.2. Read V2 codes: 3](#_Toc230786733)

[Appendix 1.1.3. ICD 9 and 10 codes: 6](#_Toc230786734)

[Appendix 1.2. Ever smoking codes 8](#_Toc230786735)

[Appendix 1.2.1. Code selection from SDEC 8](#_Toc230786736)

[Appendix 1.2.2. Read V2 codes: 8](#_Toc230786737)

[Appendix 1.3. Obesity 14](#_Toc230786738)

[Appendix 1.3.1. Code selection from SDEC 14](#_Toc230786739)

[Appendix 1.3.2. Read V2 codes: 14](#_Toc230786740)

[Appendix 1.3.3. ICD 9 and 10 codes: 16](#_Toc230786741)

[Appendix 1.4. Alcohol dependence 16](#_Toc230786742)

[Appendix 1.4.1. Code selection from SDEC 16](#_Toc230786743)

[Appendix 1.4.2. Read V2 codes: 17](#_Toc230786744)

[Appendix 1.4.3. ICD 9 and 10 codes: 20](#_Toc230786745)

[Appendix 1.5. eFI Frailty index 21](#_Toc230786746)

[Appendix 1.6. Charlson index 21](#_Toc230786747)

[Appendix 1.7. Total knee replacement codes 22](#_Toc230786748)

[Appendix 2. Supplementary results 23](#_Toc230786749)

[Appendix 2.1. Figure 1. Propensity score matching covariate balance (primary analyses) 23](#_Toc230786750)

[Appendix 2.2. Figure 2. Coefficient plot from unadjusted Aalen models 23](#_Toc230786751)

[Appendix 2.3. Figure 3. Cumulative hazard plots of Alzheimer’s dementia risk after BSI from adjusted Aalen model 24](#_Toc230786752)

[Appendix 2.4. Figure 4. Cumulative hazard plots of vascular dementia risk after BSI from adjusted Aalen model 25](#_Toc230786753)

[Appendix 2.5. Figure 5. Propensity score matching covariate balance – TKR analysis 26](#_Toc230786754)

[Appendix 2.6. Figure 6. TKR coefficient plot from unadjusted Aalen models 26](#_Toc230786755)

[Appendix 2.7. Figure 7. Propensity score matching covariate balance – lung cancer analysis 27](#_Toc230786756)

[Appendix 2.8. Figure 8. Lung cancer coefficient plot from unadjusted Aalen models 28](#_Toc230786757)

[Appendix 2.9. Figure 9. Cumulative hazard plot of dementia risk after BSI by Gram-stain from adjusted Aalen model 29](#_Toc230786758)

[Appendix 2.10. Figure 10. Cumulative hazard plot of dementia risk after BSI by peak C-reactive protein (CRP) adjusted Aalen model 30](#_Toc230786759)

[Appendix 2.11. Figure 11. Cumulative hazard plot of dementia risk after BSI by peak C-reactive protein (CRP) from adjusted Aalen model 31](#_Toc230786760)

[Appendix 2.12. Table 1. Association between bloodstream infection and dementia Cox model 32](#_Toc230786761)

[Appendix 2.13. Table 2. Uncomplicated total knee replacement (TKR) propensity matched cohort demographics 33](#_Toc230786762)

[Appendix 2.14. Table 3. Lung cancer propensity matched cohort demographics 34](#_Toc230786763)

[Appendix 2.15. Table 4. Bloodstream infection cohort demographics split by Gram stain 35](#_Toc230786764)

[Appendix 2.16. Table 5. Comparison of Gram-positive and Gram-negative BSI and risk of subsequent dementia 36](#_Toc230786765)

[Appendix 2.17. Table 6. Bloodstream infection cohort demographics split by peak C-reactive protein 37](#_Toc230786766)

[Appendix 2.18. Table 7. Association between peak C-reactive protein (CRP) with subsequent dementia 38](#_Toc230786767)

# Supplementary methods

## Dementia codes

### Code selection from SDEC

We have selected codes based on from the UK Biobank algorithm and dementia validation study (unpublished) in conjunction with the WHO ICD 10 browser (apps.who.int/classifications/icd10/browse/2010/en) and the NHS Read Code Browser (<https://isd.digital.nhs.uk/trud3/user/guest/group/0/home>). We have deliberately included codes with obvious `misspelling’ (for example having a dot where none should be) or ICD 10 codes ending with ‘X’. We have added subtype classification (Alzheimer, Vascular Dementia, Fronto-temporal dementia (FTD), Dementia with Lewis Bodies (DLB) and Unspecific) to each code again based on the UK Biobank algorithm and dementia validation study. We allowed any combination of diagnostic codes, so people can appear to have several different subtypes of dementia.

All codes that were selected for classification and the total number of people with at least one of the codes are displayed in the following tables. Please be aware that frequency counts of Read V2 codes in the table do not reflect the hierarchical nature of Read V2 coding (for example, counts of E01.. do not include E011.).

### Read V2 codes:

| **subtype** | **code** | **desc** | **total_n** |
| --- | --- | --- | --- |
| Alzheimer | Eu00. | [X]Dementia in Alzheimer’s disease | 7609 |
| Alzheimer | Eu000 | [X]Dementia in Alzheimer’s disease with early onset | 223 |
| Alzheimer | Eu001 | [X]Dementia in Alzheimer’s disease with late onset | 1094 |
| Alzheimer | Eu002 | [X]Dementia in Alzheimer’s dis, atypical or mixed type | 2455 |
| Alzheimer | Eu00z | [X]Dementia in Alzheimer’s disease, unspecified | 3208 |
| Alzheimer | F110. | Alzheimer’s disease | 15549 |
| Alzheimer | F1100 | Alzheimer’s disease with early onset | 375 |
| Alzheimer | F1101 | Alzheimer’s disease with late onset | 257 |
| Alzheimer | F112. | Senile degeneration of brain | 215 |
| Alzheimer | Fyu30 | [X]Other Alzheimer’s disease | 7 |
| DLB | Eu025 | [X]Lewy body dementia | 945 |
| DLB | F116. | Lewy body disease | 475 |
| FTD | Eu020 | [X]Dementia in Pick’s disease | 67 |
| FTD | F111. | Pick’s disease | 76 |
| FTD | F118. | Frontotemporal degeneration | 64 |
| unspec | 1461. | H/O: dementia | 4932 |
| unspec | 38C13 | Assessment of psychotic and behavioural symptoms of dementia | 22 |
| unspec | 3AE3. | GDS level 4 - moderate cognitive decline | 26 |
| unspec | 3AE4. | GDS level 5 - moderately severe cognitive decline | <5 |
| unspec | 3AE5. | GDS level 6 - severe cognitive decline | 23 |
| unspec | 3AE6. | GDS level 7 - very severe cognitive decline | <5 |
| unspec | 66h.. | Dementia monitoring | 2000 |
| unspec | 6AB.. | Dementia annual review | 47177 |
| unspec | 8BM02 | Dementia medication review | 1357 |
| unspec | 8CMe0 | Dementia advance care plan | 263 |
| unspec | 8CMG2 | Review of dementia advance care plan | 139 |
| unspec | 8CMZ. | Dementia care plan | 858 |
| unspec | 8CMZ0 | Dementia care plan agreed | 504 |
| unspec | 8CMZ1 | Dementia care plan reviewed | 475 |
| unspec | 8CMZ2 | Dementia care plan declined | 26 |
| unspec | 8CMZ3 | Dementia care plan review declined | 14 |
| unspec | 8CSA. | Dementia advance care plan agreed | 49 |
| unspec | 8Hla. | Referral to dementia care advisor | 123 |
| unspec | 9hD.. | Exception reporting: dementia quality indicators | 116 |
| unspec | 9hD0. | Excepted from dementia quality indicators: Patient unsuitable | 3992 |
| unspec | 9hD1. | Excepted from dementia quality indicators: Informed dissent | 1331 |
| unspec | 9Ou.. | Dementia monitoring administration | 318 |
| unspec | 9Ou1. | Dementia monitoring first letter | 4391 |
| unspec | 9Ou2. | Dementia monitoring second letter | 1562 |
| unspec | 9Ou3. | Dementia monitoring third letter | 751 |
| unspec | 9Ou4. | Dementia monitoring verbal invite | 159 |
| unspec | 9Ou5. | Dementia monitoring telephone invite | 376 |
| unspec | A411. | Jakob-Creutzfeldt disease | 11 |
| unspec | A4110 | Sporadic Creutzfeldt-Jakob disease | 9 |
| unspec | E00.. | Senile and presenile organic psychotic conditions | 24228 |
| unspec | E000. | Uncomplicated senile dementia | 3108 |
| unspec | E001. | Presenile dementia | 472 |
| unspec | E0010 | Uncomplicated presenile dementia | 14 |
| unspec | E0011 | Presenile dementia with delirium | 17 |
| unspec | E0012 | Presenile dementia with paranoia | 36 |
| unspec | E0013 | Presenile dementia with depression | 48 |
| unspec | E001z | Presenile dementia NOS | 58 |
| unspec | E002. | Senile dementia with depressive or paranoid features | 97 |
| unspec | E0020 | Senile dementia with paranoia | 161 |
| unspec | E0021 | Senile dementia with depression | 259 |
| unspec | E002z | Senile dementia with depressive or paranoid features NOS | 22 |
| unspec | E003. | Senile dementia with delirium | 173 |
| unspec | E012. | Other alcoholic dementia | 227 |
| unspec | E0120 | Chronic alcoholic brain syndrome | 21 |
| unspec | E02y1 | Drug-induced dementia | 8 |
| unspec | E041. | Dementia in conditions EC | 608 |
| unspec | Eu012 | [X]Subcortical vascular dementia | 66 |
| unspec | Eu013 | [X]Mixed cortical and subcortical vascular dementia | 439 |
| unspec | Eu02. | [X]Dementia in other diseases classified elsewhere | 242 |
| unspec | Eu021 | [X]Dementia in Creutzfeldt-Jakob disease | 7 |
| unspec | Eu022 | [X]Dementia in Huntington’s disease | 41 |
| unspec | Eu023 | [X]Dementia in Parkinson’s disease | 723 |
| unspec | Eu02y | [X]Dementia in other specified diseases classif elsewhere | 19 |
| unspec | Eu02z | [X] Unspecified dementia | 13394 |
| unspec | Eu041 | [X]Delirium superimposed on dementia | 51 |
| unspec | Eu106 | [X]Mental and behavioural disorders due to use of alcohol: amnesic syndrome | 101 |
| unspec | Eu107 | [X]Mental and behavioural disorders due to use of alcohol: residual and late-onset psychotic disorder | 125 |
| unspec | F11x7 | Cerebral degeneration due to Jakob - Creutzfeldt disease | 9 |
| unspec | F11x9 | Cerebral degeneration in Parkinson’s disease | 9 |
| unspec | F11y2 | Corticobasal degeneration | 37 |
| Vascular | E004. | Arteriosclerotic dementia | 2427 |
| Vascular | E0040 | Uncomplicated arteriosclerotic dementia | 88 |
| Vascular | E0041 | Arteriosclerotic dementia with delirium | 6 |
| Vascular | E0042 | Arteriosclerotic dementia with paranoia | 16 |
| Vascular | E0043 | Arteriosclerotic dementia with depression | 32 |
| Vascular | E004z | Arteriosclerotic dementia NOS | 108 |
| Vascular | Eu01. | [X]Vascular dementia | 15621 |
| Vascular | Eu010 | [X]Vascular dementia of acute onset | 22 |
| Vascular | Eu011 | [X]Multi-infarct dementia | 429 |
| Vascular | Eu01y | [X]Other vascular dementia | 138 |
| Vascular | Eu01z | [X]Vascular dementia, unspecified | 543 |
| Vascular | F11x2 | Cerebral degeneration due to cerebrovascular disease | 32 |
| Vascular | F21y2 | Binswanger’s disease | 33 |

### ICD 9 and 10 codes:

| **subtype** | **code** | **desc** | **total_n** |
| --- | --- | --- | --- |
| Alzheimer | 3310 | Alzheimer s disease | 995 |
| Alzheimer | F000 | Dementia in alzheimer disease with early onset | 1319 |
| Alzheimer | F001 | Dementia in alzheimer disease with late onset | 5571 |
| Alzheimer | F002 | Dementia in alzheimer disease atypical or mixed type | 2826 |
| Alzheimer | F009 | Dementia in alzheimer disease unspecified | 15617 |
| Alzheimer | G300 | Alzheimer disease with early onset | 1042 |
| Alzheimer | G301 | Alzheimer disease with late onset | 3223 |
| Alzheimer | G308 | Other alzheimer disease | 2437 |
| Alzheimer | G309 | Alzheimer disease unspecified | 29791 |
| FTD | 3311 | Pick s disease | <5 |
| FTD | F020 | Dementia in pick disease | 485 |
| FTD | G310 | Circumscribed brain atrophy | 804 |
| unspec | 0461 | Jakob-creutzfeldt disease | 5 |
| unspec | 2900 | Senile dementia simple type | 1220 |
| unspec | 2901 | Presenile dementia | 213 |
| unspec | 2902 | Senile dementia depressed or paranoid type | 119 |
| unspec | 2903 | Senile dementia with acute confusional state | <5 |
| unspec | 3312 | Senile degeneration of brain | 5 |
| unspec | A810 | Creutzfeldt-jakob disease | 81 |
| unspec | F021 | Dementia in creutzfeldt-jakob disease | 29 |
| unspec | F022 | Dementia in huntington disease | 85 |
| unspec | F023 | Dementia in parkinson disease | 2466 |
| unspec | F024 | Dementia in human immunodeficiency virus [hiv] disease | 5 |
| unspec | F028 | Dementia in other specified diseases classified elsewhere | 1711 |
| unspec | F03 | Unspecified dementia | 26572 |
| unspec | F03. | NA | 19 |
| unspec | F030 | NA | 13956 |
| unspec | F031 | NA | <5 |
| unspec | F033 | NA | <5 |
| unspec | F034 | NA | <5 |
| unspec | F039 | NA | <5 |
| unspec | F03X | NA | 64301 |
| unspec | F051 | Delirium superimposed on dementia | 1628 |
| unspec | F106 | Mental and behavioural disorders due to use of alcohol | 672 |
| unspec | G311 | Senile degeneration of brain not elsewhere classified | 461 |
| unspec | G318 | Other specified degenerative diseases of nervous system | 2695 |
| Vascular | 2904 | Arteriosclerotic dementia | 424 |
| Vascular | F010 | Vascular dementia of acute onset | 765 |
| Vascular | F011 | Multi-infarct dementia | 5251 |
| Vascular | F012 | Subcortical vascular dementia | 220 |
| Vascular | F013 | Mixed cortical and subcortical vascular dementia | 458 |
| Vascular | F018 | Other vascular dementia | 380 |
| Vascular | F019 | Vascular dementia unspecified | 23958 |
| Vascular | I673 | Progressive vascular leukoencephalopathy | 66 |

## Ever smoking codes

### Code selection from SDEC

We have selected codes for either based on Stocks, J et al. Examining variations in prescribing safety in UK general practice: a cross-sectional study using the Clinical Practice Research Datalink. BMJ 2015;351:h5501; Fairhurst, C et al. Sodium channel-inhibiting drugs and cancer survival: protocol for a cohort study using the CPRD primary care database. BMJ Open 2016;6:e011661; Springate, D et al. Can analyses of electronic patient records be independently and externally validated? Study 2: the Effect of Beta-Adrenoceptor Blocker Therapy on Cancer Survival; a Retrospective Cohort Study. BMJ Open 2015;5:e007299 in conjunction with the WHO ICD 10 browser (apps.who.int/classifications/icd10/browse/2010/en) and the NHS Read Code Browser (<https://isd.digital.nhs.uk/trud3/user/guest/group/0/home>). We have deliberately included codes with obvious `misspelling’ (for example having a dot where none should be) or ICD 10 codes ending with ‘X’.

Note: There are no ICD-10 codes for smoking - they only appear in the primary care data. Smoking was added to the Quality Outcomes Framework (QOF) in 2006, which may explain the sudden increase in coding around this time. Given the retrospective, observational nature of the data, we do not try to distinguish between ‘past smoking’ and ‘current smoking’.

All codes that were selected for classification and the total number of people with at least one of the codes are displayed in the following tables. Please be aware that frequency counts of Read V2 codes in the table do not reflect the hierarchical nature of Read V2 coding (for example, counts of E01.. do not include E011.).

### Read V2 codes:

| **code** | **desc** | **total_n** |
| --- | --- | --- |
| 137.. | Tobacco consumption | 114320 |
| 1372. | Trivial smoker - < 1 cig/day | 22183 |
| 1373. | Light smoker - 1-9 cigs/day | 73707 |
| 1374. | Moderate smoker - 10-19 cigs/d | 105608 |
| 1375. | Heavy smoker - 20-39 cigs/day | 65980 |
| 1376. | Very heavy smoker - 40+cigs/d | 6825 |
| 1377. | Ex-trivial smoker (<1/day) | 17793 |
| 1378. | Ex-light smoker (1-9/day) | 58529 |
| 1379. | Ex-moderate smoker (10-19/day) | 101232 |
| 137a. | Pipe tobacco consumption | 2008 |
| 137A. | Ex-heavy smoker (20-39/day) | 57813 |
| 137b. | Ready to stop smoking | 5590 |
| 137B. | Ex-very heavy smoker (40+/day) | 14902 |
| 137c. | Thinking about stopping smoking | 8318 |
| 137C. | Keeps trying to stop smoking | 2132 |
| 137d. | Not interested in stopping smoking | 12264 |
| 137D. | Admitted tobacco cons untrue ? | 53 |
| 137e. | Smoking restarted | 385 |
| 137E. | Tobacco consumption unknown | 4249 |
| 137f. | Reason for restarting smoking | 28 |
| 137F. | Ex-smoker - amount unknown | 69222 |
| 137g. | Cigarette pack-years | 5520 |
| 137G. | Trying to give up smoking | 33582 |
| 137h. | Minutes from waking to first tobacco consumption | 182 |
| 137H. | Pipe smoker | 12336 |
| 137j. | Ex-cigarette smoker | 19150 |
| 137J. | Cigar smoker | 14076 |
| 137K. | Stopped smoking | 103495 |
| 137K0 | Recently stopped smoking | 1326 |
| 137l. | Ex roll-up cigarette smoker | 870 |
| 137m. | Failed attempt to stop smoking | 2710 |
| 137M. | Rolls own cigarettes | 20862 |
| 137n. | Total time smoked | 9078 |
| 137N. | Ex pipe smoker | 5082 |
| 137o. | Waterpipe tobacco consumption | 61 |
| 137O. | Ex cigar smoker | 3725 |
| 137P. | Cigarette smoker | 205518 |
| 137Q. | Smoking started | 2550 |
| 137R. | Current smoker | 125863 |
| 137S. | Ex smoker | 429585 |
| 137T. | Date ceased smoking | 23162 |
| 137V. | Smoking reduced | 1302 |
| 137X. | Cigarette consumption | 6035 |
| 137Y. | Cigar consumption | 1278 |
| 137Z. | Tobacco consumption NOS | 14715 |
| 13p.. | Smoking cessation milestones | 4779 |
| 13p0. | Negotiated date for cessation of smoking | 1617 |
| 13p1. | Smoking status at 4 weeks | 423 |
| 13p2. | Smoking status between 4 and 52 weeks | 194 |
| 13p3. | Smoking status at 52 weeks | 16 |
| 13p4. | Smoking free weeks | 657 |
| 13p5. | Smoking cessation programme start date | 824 |
| 13p50 | Practice based smoking cessation programme start date | 272 |
| 13p6. | Carbon monoxide reading at 4 weeks | 196 |
| 13p7. | Smoking status at 12 weeks | 24 |
| 13p8. | Lost to smoking cessation follow-up | 14 |
| 38DH. | Fagerstrom test for nicotine dependence | 5 |
| 67H1. | Lifestyle advice regarding smoking | 12047 |
| 67H6. | Brief intervention for smoking cessation | 4144 |
| 745H. | Smoking cessation therapy | 9664 |
| 745H0 | Nicotine replacement therapy using nicotine patches | 1704 |
| 745H1 | Nicotine replacement therapy using nicotine gum | 207 |
| 745H2 | Nicotine replacement therapy using nicotine inhalator | 557 |
| 745H3 | Nicotine replacement therapy using nicotine lozenges | 158 |
| 745H4 | Smoking cessation drug therapy | 1264 |
| 745H5 | Varenicline therapy | 8 |
| 745Hy | Other specified smoking cessation therapy | 470 |
| 745Hz | Smoking cessation therapy NOS | 819 |
| 8B2B. | Nicotine replacement therapy | 12364 |
| 8B2B0 | Issue of nicotine replacement therapy voucher | 0 |
| 8B31G | Varenicline smoking cessation therapy offered | <5 |
| 8B3f. | Nicotine replacement therapy provided free | 2941 |
| 8B3Y. | Over the counter nicotine replacement therapy | 1157 |
| 8BP3. | Nicotine replacement therapy provided by community pharmacist | 9 |
| 8CAg. | Smoking cessation advice provided by community pharmacist | 184 |
| 8CAL. | Smoking cessation advice | 342254 |
| 8CdB. | Stop smoking service opportunity signposted | 1965 |
| 8H7i. | Referral to smoking cessation advisor | 14754 |
| 8HBM. | Stop smoking face to face follow-up | 291 |
| 8HBP. | Smoking cessation 12 week follow-up | 10 |
| 8HkQ. | Referral to NHS stop smoking service | 5839 |
| 8HTK. | Referral to stop-smoking clinic | 11965 |
| 8I2I. | Nicotine replacement therapy contraindicated | <5 |
| 8I39. | Nicotine replacement therapy refused | 725 |
| 8IAj. | Smoking cessation advice declined | 20230 |
| 8IEK. | Smoking cessation programme declined | 7603 |
| 8IEM. | Smoking cessation drug therapy declined | 8779 |
| 8IEM0 | Varenicline smoking cessation therapy declined | 0 |
| 8IEo. | Referral to smoking cessation service declined | 4017 |
| 8T08. | Referral to smoking cessation service | 745 |
| 9hG.. | Exception reporting: smoking quality indicators | 626 |
| 9hG0. | Excepted from smoking quality indicators: Patient unsuitable | 5297 |
| 9hG1. | Excepted from smoking quality indicators: Informed dissent | 10264 |
| 9kc.. | Smoking cessation - enhanced services administration | 83 |
| 9kc0. | Smoking cessation monitoring template completed - enhanced services administration | <5 |
| 9kf1. | Referred for chronic obstructive pulmonary disease structured smoking assessment - enhanced services administration | <5 |
| 9kf2. | Chronic obstructive pulmonary disease structured smoking assessment declined - enhanced services administration | 10 |
| 9km.. | Ex-smoker annual review - enhanced services administration | 508 |
| 9ko.. | Current smoker annual review - enhanced services administration | 446 |
| 9N2k. | Seen by smoking cessation advisor | 4489 |
| 9N4M. | DNA - Did not attend smoking cessation clinic | 887 |
| 9Ndf. | Consent given for follow-up by smoking cessation team | 219 |
| 9Ndg. | Declined consent for follow-up by smoking cessation team | 2658 |
| 9NdV. | Consent given for follow-up evaluation after smoking cessation intervention | 6 |
| 9NdW. | Consent given for smoking cessation data sharing | 81 |
| 9NdY. | Declined consent for follow-up evaluation after smoking cessation intervention | 58 |
| 9NdZ. | Declined consent for smoking cessation data sharing | 82 |
| 9NS02 | Referral for smoking cessation service offered | 3786 |
| 9OO.. | Anti-smoking monitoring admin. | 3791 |
| 9OO1. | Attends stop smoking monitor. | 1268 |
| 9OO2. | Refuses stop smoking monitor | 1239 |
| 9OO3. | Stop smoking monitor default | 168 |
| 9OO4. | Stop smoking monitor 1st lettr | 7636 |
| 9OO5. | Stop smoking monitor 2nd lettr | 1622 |
| 9OO6. | Stop smoking monitor 3rd lettr | 658 |
| 9OO7. | Stop smoking monitor verb.inv. | 1418 |
| 9OO8. | Stop smoking monitor phone inv | 615 |
| 9OO9. | Stop smoking monitoring delete | 6 |
| 9OOA. | Stop smoking monitor.chck done | 554 |
| 9OOB. | Stop smoking invitation short message service text message | <5 |
| 9OOB0 | Stop smoking invitation first short message service text message | 49 |
| 9OOB1 | Stop smoking invitation second short message service text message | <5 |
| 9OOB2 | Stop smoking invitation third short message service text message | 5 |
| 9OOZ. | Stop smoking monitor admin.NOS | 366 |
| E023. | Nicotine withdrawal | 120 |
| E251. | Tobacco dependence | 1802 |
| E2510 | Tobacco dependence, unspecified | <5 |
| E2511 | Tobacco dependence, continuous | <5 |
| E2512 | Tobacco dependence, episodic | 0 |
| E2513 | Tobacco dependence in remission | 0 |
| E251z | Tobacco dependence NOS | <5 |
| Eu17. | [X]Mental and behavioural disorder due to use of tobacco | 0 |
| Eu170 | [X]Mental and behavioural disorders due to use of tobacco: acute intoxication | <5 |
| Eu171 | [X]Mental and behavioural disorders due to use of tobacco: harmful use | 5 |
| Eu172 | [X]Mental and behavioural disorders due to use of tobacco: dependence syndrome | 0 |
| Eu173 | [X]Mental and behavioural disorders due to use of tobacco: withdrawal state | 0 |
| Eu174 | [X]Mental and behavioural disorders due to use of tobacco: withdrawal state with delirium | 0 |
| Eu175 | [X]Mental and behavioural disorders due to use of tobacco: psychotic disorder | 0 |
| Eu176 | [X]Mental and behavioural disorders due to use of tobacco: amnesic syndrome | 0 |
| Eu177 | [X]Mental and behavioural disorders due to use of tobacco: residual and late-onset psychotic disorder | 0 |
| Eu17y | [X]Mental and behavioural disorders due to use of tobacco: other mental and behavioural disorders | 0 |
| Eu17z | [X]Mental and behavioural disorders due to use of tobacco: unspecified mental and behavioural disorder | 0 |
| H3101 | Smokers’ cough | 831 |
| J0364 | Tobacco deposit on teeth | 0 |
| J0873 | Leukokeratosis nicotina palati | 6 |
| SMC.. | Toxic effect of tobacco and nicotine | 5 |
| ZV116 | [V]Personal history of tobacco abuse | <5 |
| ZV4K0 | [V]Tobacco use | 311 |
| ZV6D8 | [V]Tobacco abuse counselling | <5 |
| 1370. | NA | <5 |
| 137H0 | NA | 31 |
| 137j0 | NA | 98 |
| 137J0 | NA | 34 |
| 137M0 | NA | <5 |
| 137n0 | NA | <5 |
| 137N0 | NA | <5 |
| 137O0 | NA | <5 |
| 137P0 | NA | 17 |
| 137R0 | NA | <5 |
| 137s. | NA | 262 |
| 137S0 | NA | 5 |
| 137T0 | NA | <5 |
| 137Y0 | NA | <5 |

## Obesity codes

### Code selection from SDEC

We have selected codes based on Doran, T et al. Effect of financial incentives on incentivised and non-incentivised clinical activities: longitudinal analysis of data from the UK Quality and Outcomes Framework. British Medical Journal. BMJ 2011; 342:d3590; Reeves, D et al. Can analyses of electronic patient records be independently and externally validated? The effect of statins on the mortality of patients with Ischaemic heart disease: a cohort study with nested case-control analysis. BMJ Open 2014; 4:e004952; Fairhurst, C et al. Exposure to sodium channel-inhibiting drugs and cancer survival: protocol for a cohort study using the QResearch primary care database. BMJ Open 2016;6:e011661; in conjunction with the WHO ICD 10 browser (apps.who.int/classifications/icd10/browse/2010/en) and the NHS Read Code Browser (<https://isd.digital.nhs.uk/trud3/user/guest/group/0/home>). We have deliberately included codes with obvious `misspelling’ (for example having a dot where none should be) or ICD 10 codes ending with ‘X’.

All codes that were selected for classification and the total number of people with at least one of the codes are displayed in the following tables. Please be aware that frequency counts of Read V2 codes in the table do not reflect the hierarchical nature of Read V2 coding (for example, counts of E01.. do not include E011.).

### Read V2 codes:

| **code** | **desc** | **total_n** |
| --- | --- | --- |
| 22A5. | O/E - weight > 20% over ideal | 2488 |
| 22K5. | Body mass index 30+ - obesity | 49947 |
| 22K7. | Body mass index 40+ - severely obese | 5184 |
| 38Qb. | National Obesity Observatory Standard Evaluation Framework for weight management interventions - participant satisfaction with intervention | 6 |
| 66C1. | Initial obesity assessment | 773 |
| 66C2. | Follow-up obesity assessment | 1088 |
| 66C4. | Has seen dietician - obesity | 728 |
| 66C5. | Treatment of obesity changed | 27 |
| 66C6. | Treatment of obesity started | 283 |
| 66C7. | Treatment of obesity stopped | 47 |
| 66Cb. | Intensive weight management programme commenced | <5 |
| 66Cc. | Intensive weight management programme ended | <5 |
| 66Cd. | Intensive weight management programme declined | 14 |
| 66CE. | Reason for obesity therapy - occupational | 25 |
| 66CL. | Risk to health associated with overweight and obesity, at no increased risk | 0 |
| 66CM. | Risk to health associated with overweight and obesity, at increased risk | <5 |
| 66CN. | Risk to health associated with overweight and obesity, at high risk | <5 |
| 66CP. | Risk to health associated with overweight and obesity, at very high risk | 26 |
| 66CQ. | Intervention for risk to health associated with overweight and obesity, general advice on healthy weight and lifestyle | 879 |
| 66CR. | Intervention for risk to health associated with overweight and obesity, advice about diet and physical activity | 100 |
| 66CS. | Intervention for risk to health associated with overweight and obesity, advice about diet and physical activity, consider drugs | 0 |
| 66CT. | Intervention for risk to health associated with overweight and obesity, advice about diet and physical activity, consider drugs, consider surgery | 0 |
| 66CW. | Unsuitable for weight management programme | 20 |
| 66CX. | Obesity multidisciplinary case review | 0 |
| 66CZ. | Obesity monitoring NOS | 941 |
| 8CT5. | Anti-obesity drug therapy discontinued | <5 |
| 8CV7. | Anti-obesity drug therapy commenced | <5 |
| 8T11. | Referral to multidisciplinary obesity clinic | 5 |
| 9hN.. | Exception reporting: obesity quality indicators | 0 |
| 9hN0. | Excepted from obesity quality indicators: patient unsuitable | 13 |
| 9hN1. | Excepted from obesity quality indicators: informed dissent | <5 |
| 9OK.. | Obesity monitoring admin. | 273 |
| 9OK1. | Attends obesity monitoring | 445 |
| 9OK2. | Refuses obesity monitoring | 96 |
| 9OK3. | Obesity monitoring default | 28 |
| 9OK4. | Obesity monitoring 1st letter | 818 |
| 9OK5. | Obesity monitoring 2nd letter | 152 |
| 9OK6. | Obesity monitoring 3rd letter | 72 |
| 9OK7. | Obesity monitoring verbal inv. | 14 |
| 9OK8. | Obesity monitor phone invite | 25 |
| 9OK9. | Obesity monitoring deleted | 0 |
| 9OKA. | Obesity monitoring check done | 1286 |
| 9OKZ. | Obesity monitoring admin.NOS | 7 |
| C380. | Obesity | 42303 |
| C3800 | Obesity due to dependence calories | 99 |
| C3801 | Drug-induced obesity | 9 |
| C3802 | Extreme obesity with alveolar hypoventilation | 77 |
| C3803 | Morbid obesity | 1986 |
| C3804 | Central obesity | 555 |
| C3805 | Generalised obesity | 112 |
| C3806 | Adult-onset obesity | 0 |
| C3807 | Lifelong obesity | 0 |
| C3808 | Childhood obesity | 0 |
| C38y0 | Pickwickian syndrome | 271 |
| C38z0 | Simple obesity NOS | 363 |
| ZV653 | [V]Dietary surveillance and counselling | 6804 |

### ICD 9 and 10 codes:

| **code** | **desc** | **total_n** |
| --- | --- | --- |
| 2780 | Obesity | 104 |
| E66 | Obesity | 0 |
| E660 | Obesity due to dependence calories | 110 |
| E661 | Drug-induced obesity | 15 |
| E662 | Extreme obesity with alveolar hypoventilation | 857 |
| E668 | Other obesity | 4646 |
| E669 | Obesity unspecified | 35498 |
| E66X | NA | <5 |

## Alcohol dependence codes

### Code selection from SDEC

We have selected codes based on Thompson, A et al. Drug therapy for alcohol dependence in primary care in the UK: A Clinical Practice Research Datalink study. PLoS ONE. 2017; 12(3) e0173272 and Carr MJ et al. Premature death among primary care patients with a history of self-harm. Ann Fam Med. 2017; 15(3): 246-254 (list at <https://clinicalcodes.rss.mhs.man.ac.uk/medcodes/article/41>) in conjunction with the WHO ICD 10 browser (apps.who.int/classifications/icd10/browse/2010/en) and the NHS Read Code Browser (<https://isd.digital.nhs.uk/trud3/user/guest/group/0/home>). We have deliberately included codes with obvious `misspelling’ (for example having a dot where none should be) or ICD 10 codes ending with ‘X’.

All codes that were selected for classification and the total number of people with at least one of the codes are displayed in the following tables. Please be aware that frequency counts of Read V2 codes in the table do not reflect the hierarchical nature of Read V2 coding (for example, counts of E01.. do not include E011.).

### Read V2 codes:

| **code** | **desc** | **total_n** |
| --- | --- | --- |
| 1366. | Very heavy drinker - >9u/day | 7407 |
| 136Q. | Very heavy drinker | 703 |
| 136T. | Harmful alcohol use | 1082 |
| 1462. | H/O: alcoholism | 1706 |
| 1B1c. | Alcohol induced hallucinations | 39 |
| 66e0. | Alcohol abuse monitoring | 449 |
| 7P221 | Delivery of rehabilitation for alcohol addiction | 22 |
| 8BA8. | Alcohol detoxification | 2663 |
| 8CAv. | Advised to contact primary care alcohol worker | 13 |
| 8G32. | Aversion therapy - alcoholism | 52 |
| 8H35. | Admitted to alcohol detoxification centre | 221 |
| 8HkG. | Referral to specialist alcohol treatment service | 57 |
| 8IAF. | Brief intervention for dependenceive alcohol consumption declined | 53 |
| 8IAJ. | Declined referral to specialist alcohol treatment service | 97 |
| 8IAt. | Extended intervention for dependenceive alcohol consumption declined | 20 |
| 9k1B. | Extended intervention for dependenceive alcohol consumption completed | 6 |
| 9NN2. | Under care of community alcohol team | 176 |
| C1505 | Alcohol-induced pseudo-Cushing’s syndrome | <5 |
| E01.. | Alcoholic psychoses | 99 |
| E010. | Alcohol withdrawal delirium | 355 |
| E011. | Alcohol amnestic syndrome | 20 |
| E0110 | Korsakov’s alcoholic psychosis | 239 |
| E0111 | Korsakov’s alcoholic psychosis with peripheral neuritis | 14 |
| E0112 | Wernicke-Korsakov syndrome | 90 |
| E011z | Alcohol amnestic syndrome NOS | <5 |
| E012. | Other alcoholic dementia | 227 |
| E0120 | Chronic alcoholic brain syndrome | 21 |
| E013. | Alcohol withdrawal hallucinosis | 27 |
| E014. | Pathological alcohol intoxication | 51 |
| E015. | Alcoholic paranoia | 15 |
| E01y. | Other alcoholic psychosis | 67 |
| E01y0 | Alcohol withdrawal syndrome | 1703 |
| E01yz | Other alcoholic psychosis NOS | <5 |
| E01z. | Alcoholic psychosis NOS | 26 |
| E23.. | Alcohol dependence syndrome | 18360 |
| E230. | Acute alcoholic intoxication in alcoholism | 499 |
| E2300 | Acute alcoholic intoxication, unspecified, in alcoholism | 33 |
| E2301 | Continuous acute alcoholic intoxication in alcoholism | 9 |
| E2302 | Episodic acute alcoholic intoxication in alcoholism | 13 |
| E2303 | Acute alcoholic intoxication in remission, in alcoholism | <5 |
| E230z | Acute alcoholic intoxication in alcoholism NOS | 41 |
| E231. | Chronic alcoholism | 776 |
| E2310 | Unspecified chronic alcoholism | 47 |
| E2311 | Continuous chronic alcoholism | 212 |
| E2312 | Episodic chronic alcoholism | 137 |
| E2313 | Chronic alcoholism in remission | 135 |
| E231z | Chronic alcoholism NOS | 197 |
| E23z. | Alcohol dependence syndrome NOS | 879 |
| E2503 | Nondependent alcohol abuse in remission | 89 |
| Eu10. | [X]Mental and behavioural disorders due to use of alcohol | 106 |
| Eu100 | [X]Mental and behavioural disorders due to use of alcohol: acute intoxication | 143 |
| Eu101 | [X]Mental and behavioural disorders due to use of alcohol: harmful use | 59 |
| Eu102 | [X]Mental and behavioural disorders due to use of alcohol: dependence syndrome | 563 |
| Eu103 | [X]Mental and behavioural disorders due to use of alcohol: withdrawal state | 108 |
| Eu104 | [X]Mental and behavioural disorders due to use of alcohol: withdrawal state with delirium | 104 |
| Eu105 | [X]Mental and behavioural disorders due to use of alcohol: psychotic disorder | 93 |
| Eu106 | [X]Mental and behavioural disorders due to use of alcohol: amnesic syndrome | 101 |
| Eu107 | [X]Mental and behavioural disorders due to use of alcohol: residual and late-onset psychotic disorder | 125 |
| Eu108 | [X]Alcohol withdrawal-induced seizure | 227 |
| Eu10y | [X]Mental and behavioural disorders due to use of alcohol: other mental and behavioural disorders | <5 |
| Eu10z | [X]Mental and behavioural disorders due to use of alcohol: unspecified mental and behavioural disorder | <5 |
| F11x0 | Cerebral degeneration due to alcoholism | 121 |
| F1440 | Cerebellar ataxia due to alcoholism | 43 |
| F25B. | Alcohol-induced epilepsy | 48 |
| F375. | Alcoholic polyneuropathy | 170 |
| F3941 | Alcoholic myopathy | 34 |
| G555. | Alcoholic cardiomyopathy | 278 |
| G8523 | Oesophageal varices in alcoholic cirrhosis of the liver | 57 |
| J610. | Alcoholic fatty liver | 768 |
| J611. | Acute alcoholic hepatitis | 415 |
| J612. | Alcoholic cirrhosis of liver | 2208 |
| J6120 | Alcoholic fibrosis and sclerosis of liver | 20 |
| J613. | Alcoholic liver damage unspecified | 2429 |
| J6130 | Alcoholic hepatic failure | 92 |
| J617. | Alcoholic hepatitis | 387 |
| J6170 | Chronic alcoholic hepatitis | 29 |
| J6710 | Alcohol-induced chronic pancreatitis | 130 |
| SLH3. | Alcohol deterrent poisoning | 5 |
| SM001 | Denatured alcohol causing toxic effect | <5 |
| U60H3 | [X]Alcohol deterrents causing adverse effects in therapeutic use | 0 |

### ICD 9 and 10 codes:

| **code** | **desc** | **total_n** |
| --- | --- | --- |
| 291 | Alcoholic psychoses | 0 |
| 2910 | Delirium tremens | <5 |
| 2911 | Korsakov s psychosis alcoholic | 0 |
| 2912 | Other alcoholic dementia | <5 |
| 2913 | Other alcoholic hallucinosis | <5 |
| 2914 | Pathological drunkenness | 0 |
| 2915 | Alcoholic jealousy | 0 |
| 2918 | Other | 5 |
| 2919 | Unspecified | 0 |
| 303 | Alcohol dependence syndrome | 117 |
| 4255 | Alcoholic cardiomyopathy | 15 |
| 5710 | Alcoholic fatty liver | 12 |
| E244 | Alcohol-induced pseudo-Cushing syndrome | 6 |
| F10. | NA | 46 |
| F101 | Mental and behavioural disorders due to use of alcohol | 14063 |
| F102 | Mental and behavioural disorders due to use of alcohol | 15060 |
| F103 | Mental and behavioural disorders due to use of alcohol | 4561 |
| F104 | Mental and behavioural disorders due to use of alcohol | 699 |
| F105 | Mental and behavioural disorders due to use of alcohol | 237 |
| F106 | Mental and behavioural disorders due to use of alcohol | 672 |
| F107 | Mental and behavioural disorders due to use of alcohol | 571 |
| F108 | Mental and behavioural disorders due to use of alcohol | 47 |
| F109 | Mental and behavioural disorders due to use of alcohol | 589 |
| F10U | NA | <5 |
| F10X | NA | 101 |
| G312 | Degeneration of nervous system due to alcohol | 754 |
| G721 | Alcoholic myopathy | 92 |
| I426 | Alcoholic cardiomyopathy | 548 |
| K70 | Alcoholic liver disease | 0 |
| K700 | Alcoholic fatty liver | 629 |
| K701 | Alcoholic hepatitis | 865 |
| K702 | Alcoholic fibrosis and sclerosis of liver | 68 |
| K703 | Alcoholic cirrhosis of liver | 4791 |
| K704 | Alcoholic hepatic failure | 1482 |
| K709 | Alcoholic liver disease unspecified | 7279 |
| K70D | NA | <5 |
| K70X | NA | <5 |
| K852 | Alcohol-induced acute pancreatitis | 147 |
| K860 | Alcohol-induced chronic pancreatitis | 949 |

## eFI Frailty index

The eFI is based on the internationally established cumulative deficit model and it assigns a frailty score to an individual based on 36 variables from primary care data. These include symptoms, signs, diseases, disabilities and abnormal laboratory results referred to as deficits. The eFI is the number of deficits present, expressed as an equally weighted proportion of the total. It is also a useful predictor of increased mortality in the year following incident hospital admission.

An individual with an EFI of 1 would be 1/36 (0.03) whereas a person with a score of 9 deficits would be 9/36 (0.25) .

The eFI score is then used to categorise individuals as:

- Fit ( 0 -0.12)
- Mild (>0.12 -0.24)
- Moderate (>0.24 – 0.36)
- Severely frail (>0.36)

##

## Charlson index codes

The Charlson index is based on attendances at hospital for a number of different conditions in the year before an index date. The index date taken here for the SDEC population in the 1^st^ January 2010. he Charlson Comorbidity Index for each provider spell is calculated as the sum of the weights for each of the conditions.


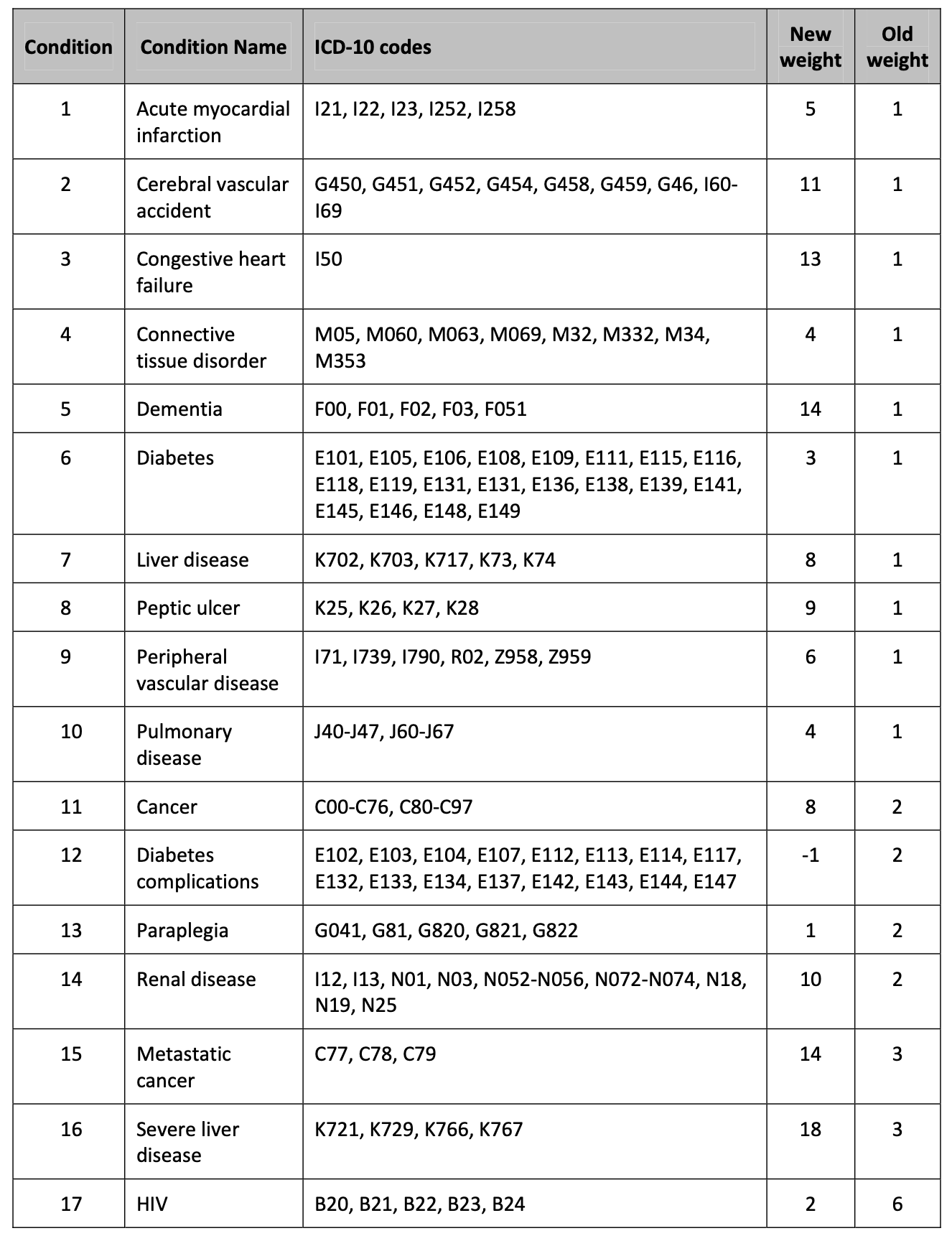


## Total knee replacement codes

OPCS4 codes: W40, W401, W41, W411, W21

Excluded those with infective complications: ICD 10 codes: T845, T846, T847

# Supplementary results

## Figure 1. Propensity score matching covariate balance (primary analyses)


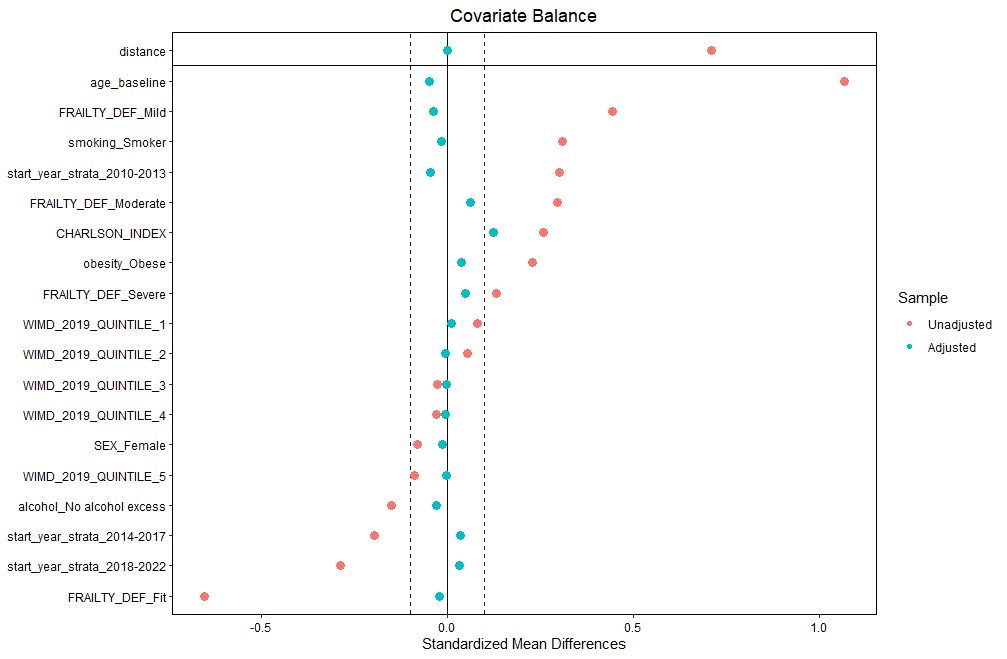


## Figure 2. Coefficient plot from unadjusted Aalen models

Uniform 95% confidence intervals to account for time-varying effect of infection


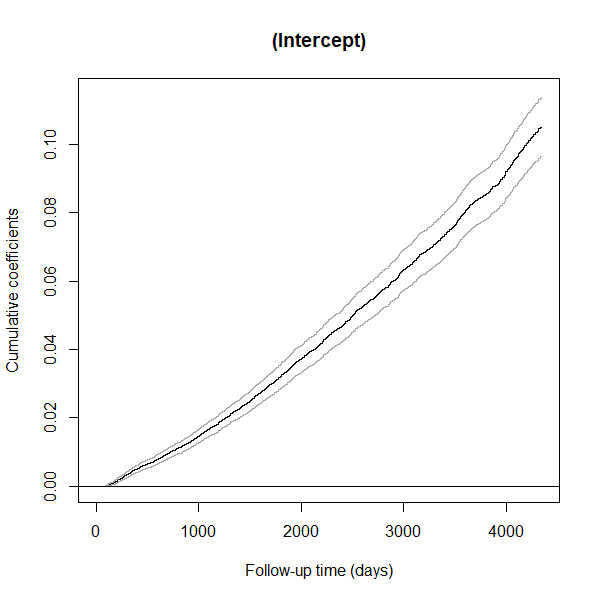

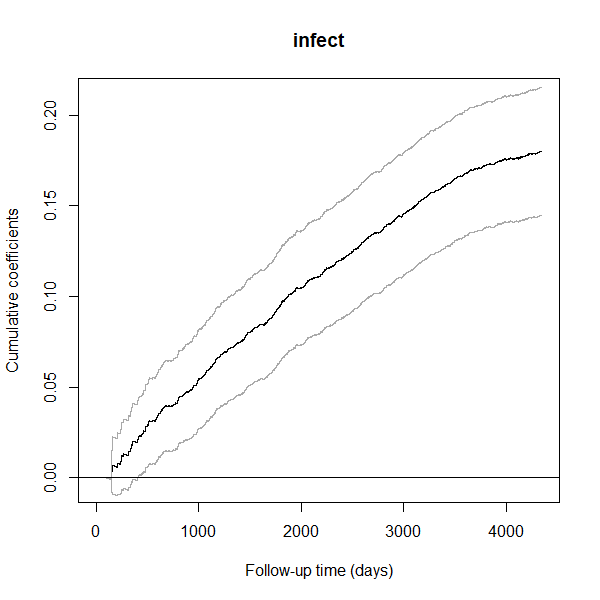


## Figure 3. Cumulative hazard plots of Alzheimer’s dementia risk after BSI from adjusted Aalen model

Uniform 95% confidence intervals to account for time-varying effect of infection


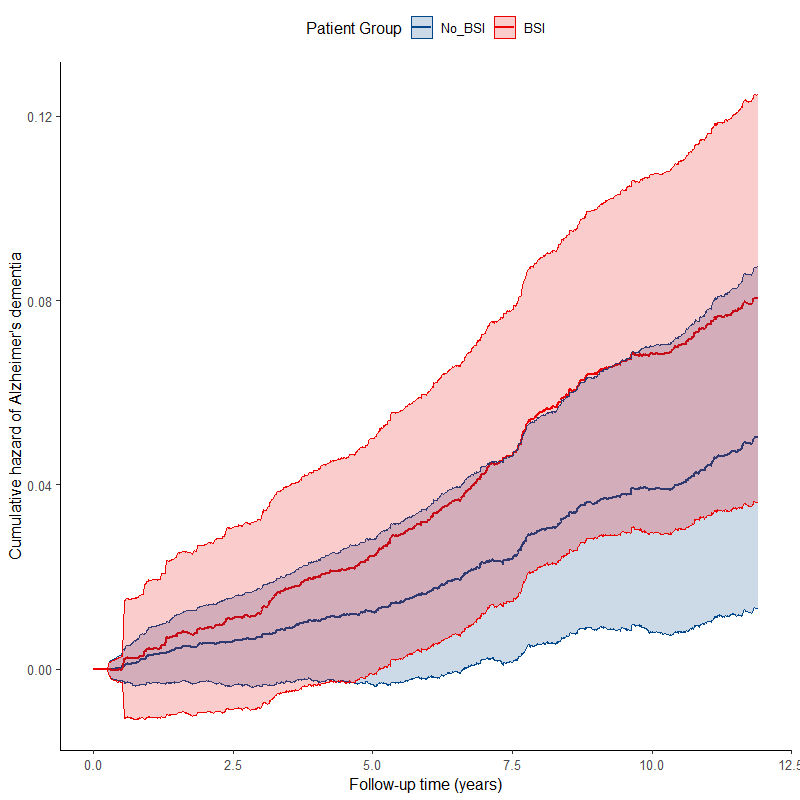


Plotted with uniform 95% confidence intervals, whereby the confidence level applies simultaneously across the entire range of the curve, not just at individual time points.

## Figure 4. Cumulative hazard plots of vascular dementia risk after BSI from adjusted Aalen model

Uniform 95% confidence intervals to account for time-varying effect of infection

**
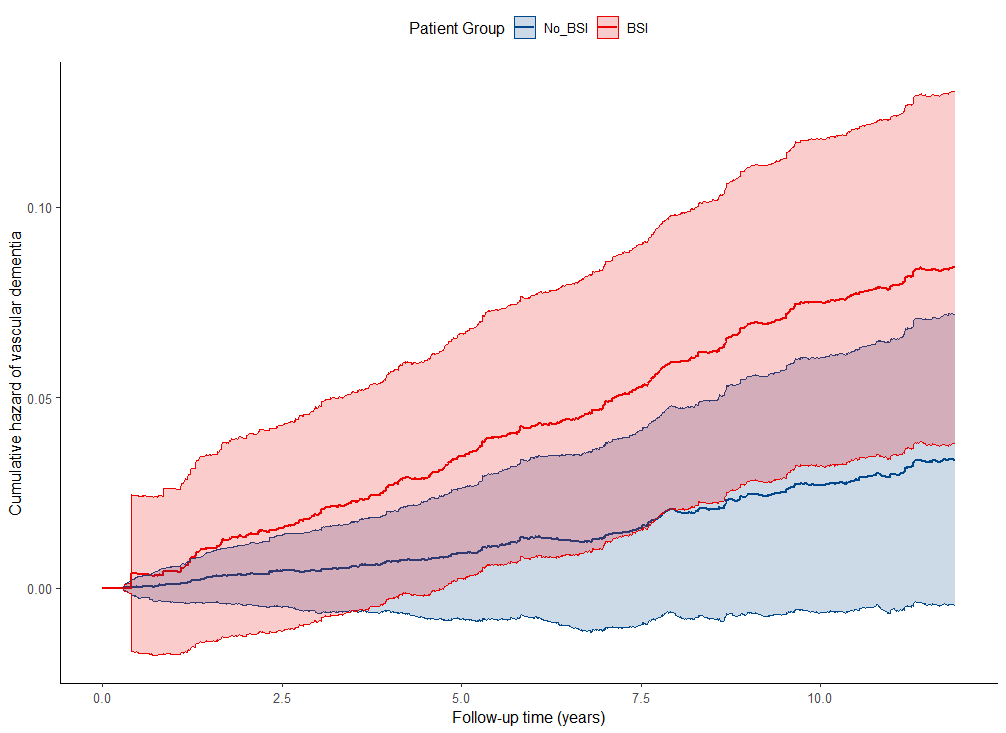
**

Plotted with uniform 95% confidence intervals, whereby the confidence level applies simultaneously across the entire range of the curve, not just at individual time points.

## Figure 5. Propensity score matching covariate balance – TKR analysis


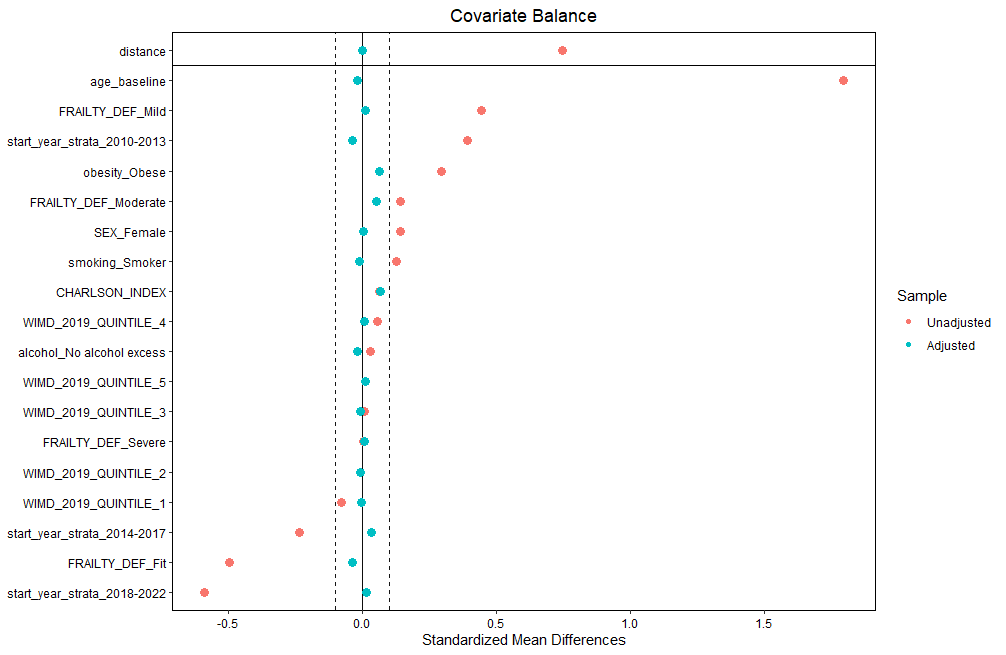


## Figure 6. TKR coefficient plot from unadjusted Aalen models

Uniform 95% confidence intervals to account for time-varying effect of infection


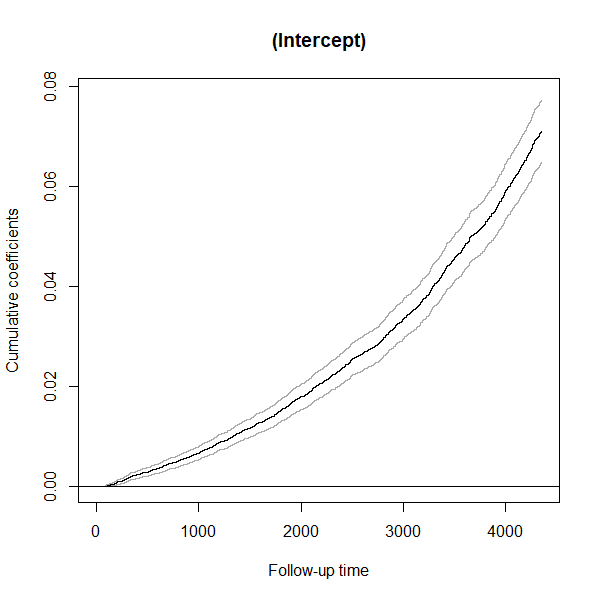

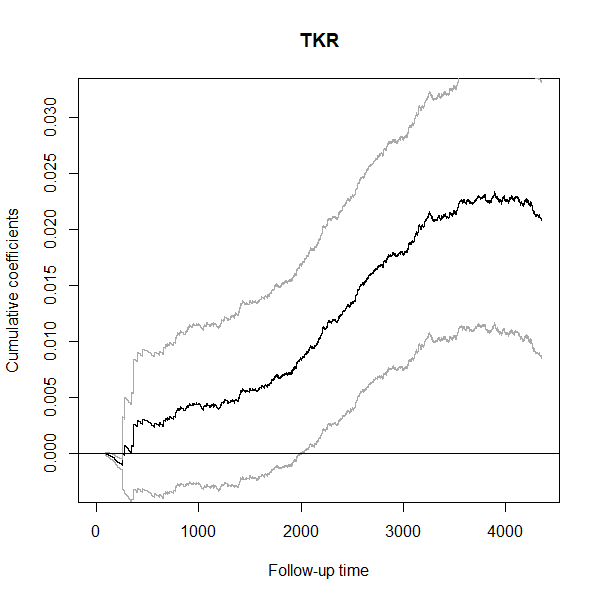


## Figure 7. Propensity score matching covariate balance – lung cancer analysis


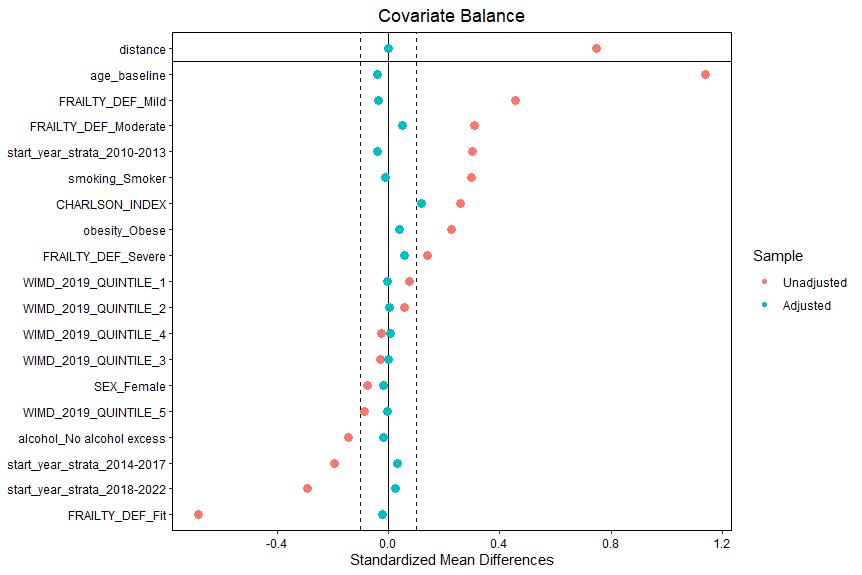


## Figure 8. Lung cancer coefficient plot from unadjusted Aalen models

Uniform 95% confidence intervals to account for time-varying effect of infection


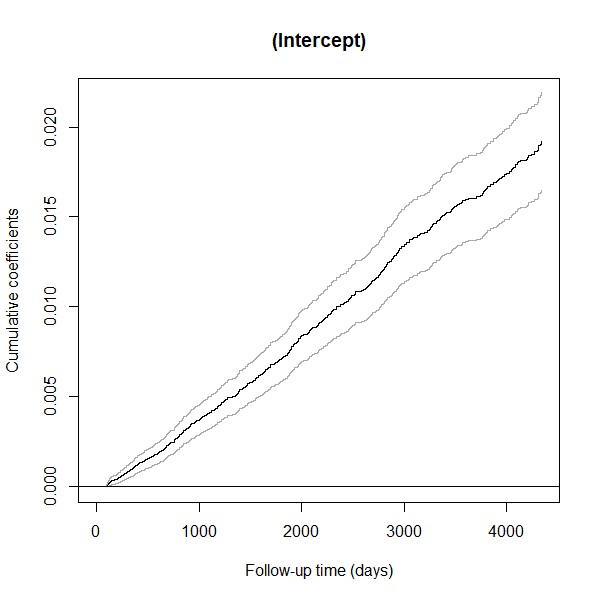

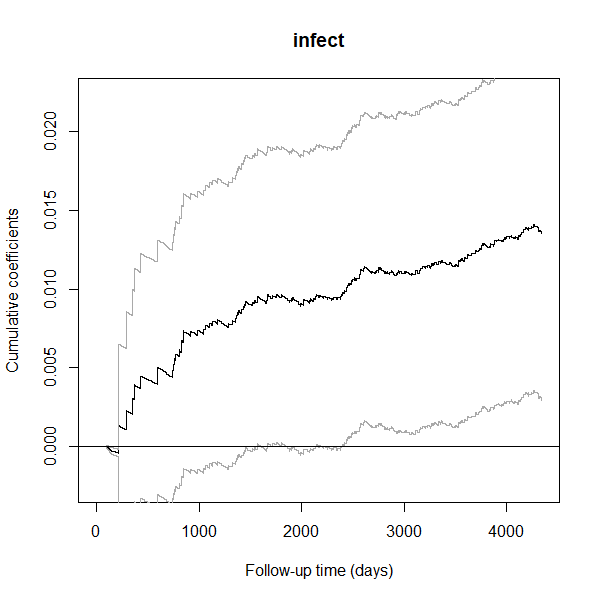


## Figure 9. Cumulative hazard plot of dementia risk after BSI by Gram-stain from adjusted Aalen model

Uniform 95% confidence intervals to account for time-varying effect of infection


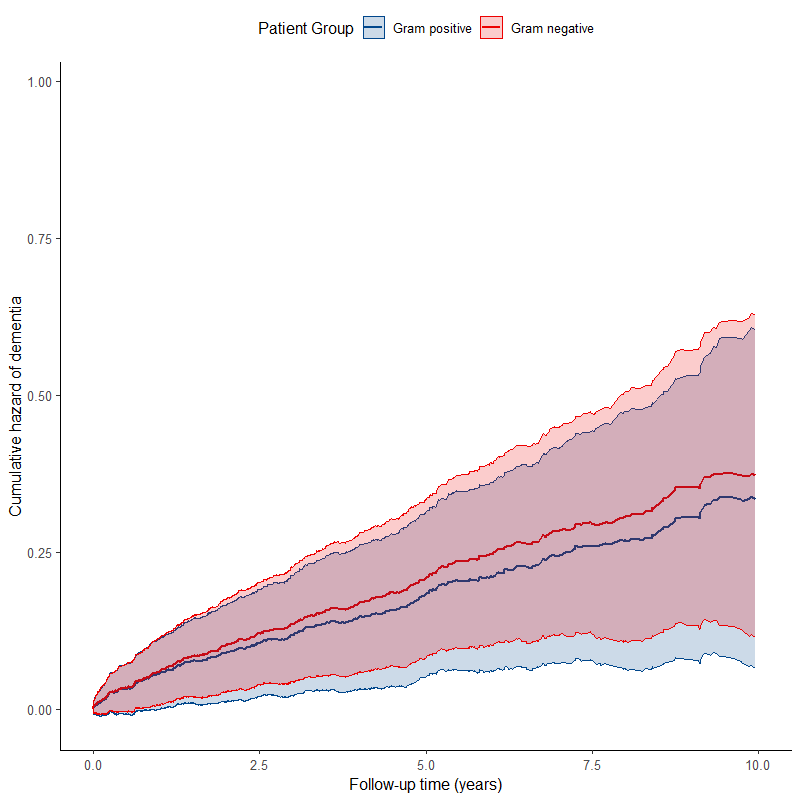


Plotted with uniform 95% confidence intervals, whereby the confidence level applies simultaneously across the entire range of the curve, not just at individual time points.

## Figure 10. Cumulative hazard plot of dementia risk after BSI by peak C-reactive protein (CRP) adjusted Aalen model

Uniform 95% confidence intervals to account for time-varying effect of infection


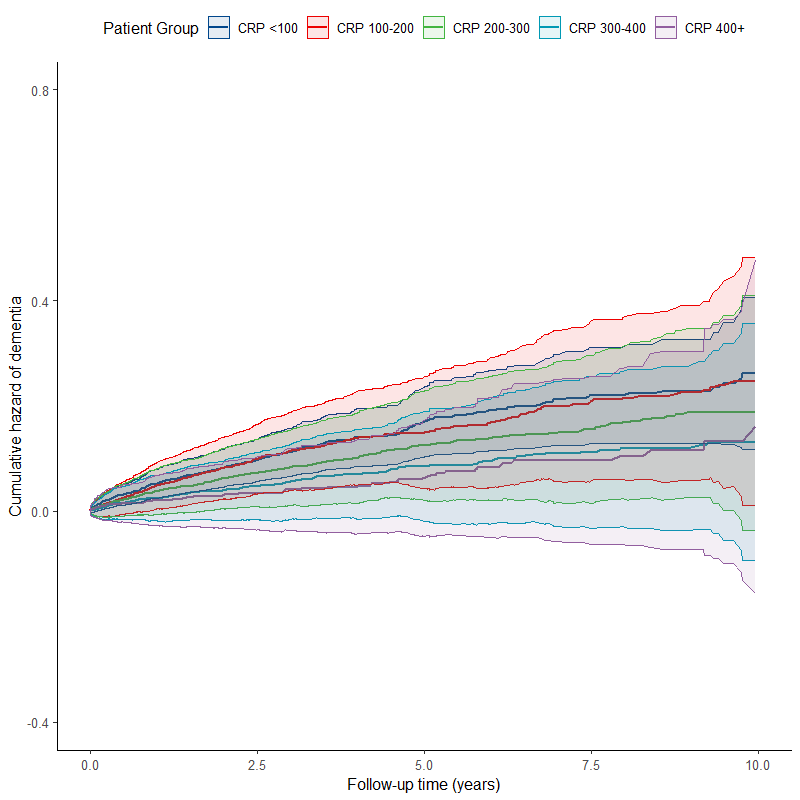


Plotted with uniform 95% confidence intervals, whereby the confidence level applies simultaneously across the entire range of the curve, not just at individual time points.

## Figure 11. Cumulative hazard plot of dementia risk after BSI by peak C-reactive protein (CRP) from adjusted Aalen model

Plotted without confidence intervals for clarity


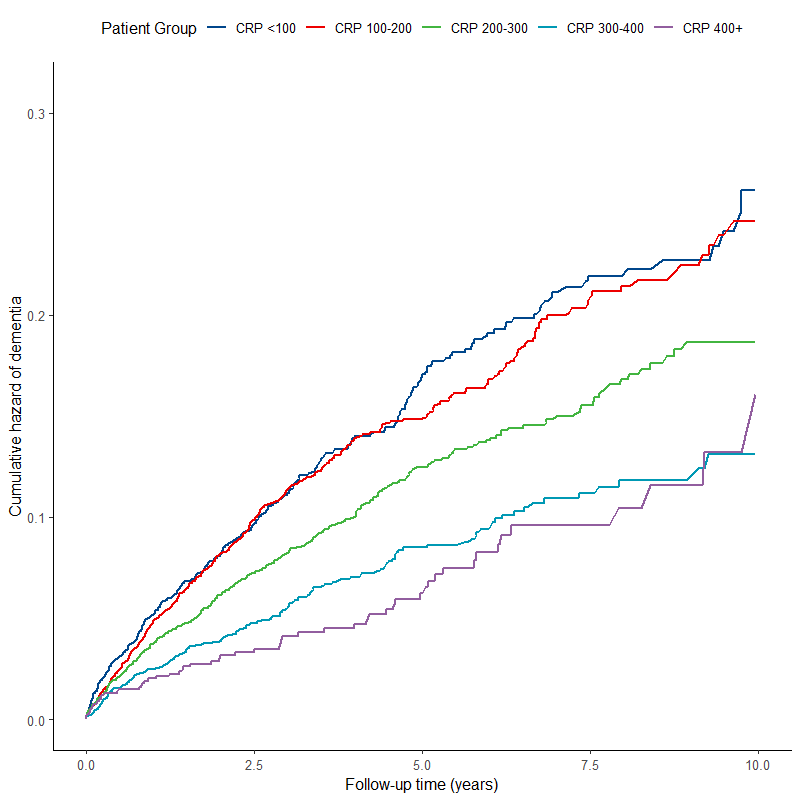


## Table 1. Association between bloodstream infection and dementia Cox model

|  | | **Unadjusted** | | | | | **Adjusted** | | | | |
| --- | --- | --- | --- | --- | --- | --- | --- | --- | --- | --- | --- |
| **Group** |  | **N** | **Event N** | **HR** | **95% CI** | **p-value** | **N** | **Event N** | **HR** | **95% CI** | **p-value** |
| Dementia | **Bloodstream infection** | 50,668 | 4,688 | 2.50 | 2.33 to 2.67 | <0.001 | 50,668 | 4,688 | 2.18 | 2.04 to 2.34 | <0.001 |
| Alzheimer's | **Bloodstream infection** | 50,668 | 1,497 | 1.76 | 1.55 to 2.00 | <0.001 | 50,668 | 1,497 | 1.61 | 1.42 to 1.83 | <0.001 |
| Vascular | **Bloodstream infection** | 50,668 | 1,478 | 2.45 | 2.17 to 2.77 | <0.001 | 50,668 | 1,478 | 2.11 | 1.87 to 2.39 | <0.001 |
| Abbreviations: CI = Confidence Interval, HR = Hazard Ratio | | | | | | | | | | | |

Models adjusted for baseline age, sex, CCI, eFI, WIMD, start year strata, obesity, alcohol use and smoking

Scaled Schonfeld residuals showing non-proportional hazard of dementia


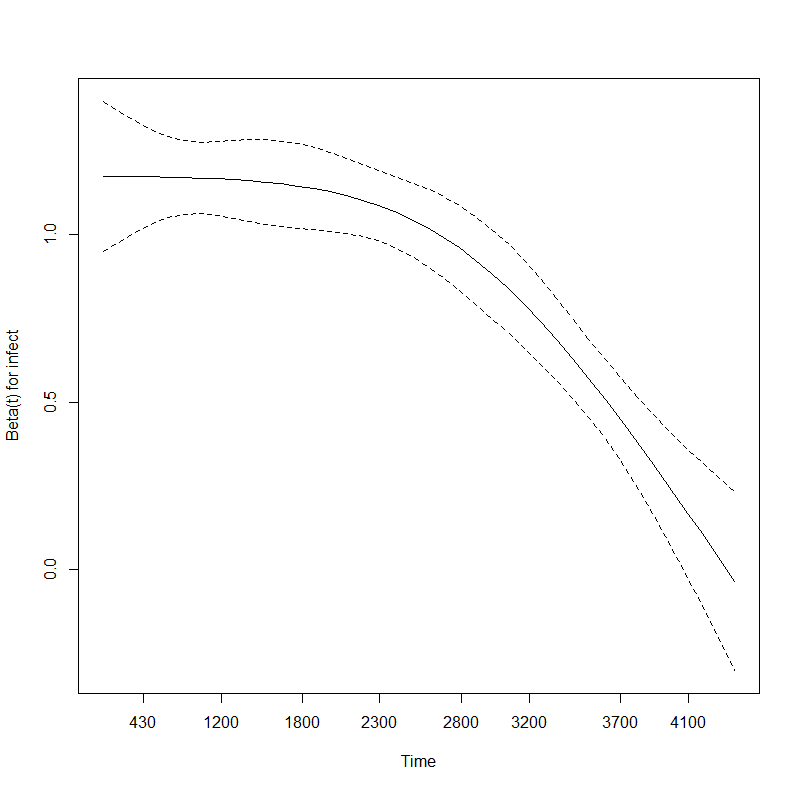


## Table 2. Uncomplicated total knee replacement (TKR) propensity matched cohort demographics

|  | **Uncomplicated TKR** | |  |
| --- | --- | --- | --- |
| **Variable** | **No TKR**  N = 21,822^1^ | **TKR**  N = 21,822^1^ | **p-value**^2^ |
| **Age (years)** | 65 (58 – 72) | 64 (58 – 71) | 0.10 |
| **Sex** |  |  | 0.57 |
| *Male* | 9,423 (43) | 9,365 (43) |  |
| *Female* | 12,399 (57) | 12,457 (57) |  |
| **Charlson Index** |  |  | 0.42 |
| *Up to 20* | 21,791 (100) | 21,798 (100) |  |
| *21-40* | 31 (0.1) | 24 (0.1) |  |
| *41-50* | 0 (0) | 0 (0) |  |
| *>50* | 0 (0) | 0 (0) |  |
| **Welsh Index of Multiple Deprivation** |  |  | 0.71 |
| *5* | 4,545 (21) | 4,629 (21) |  |
| *4* | 4,676 (21) | 4,742 (22) |  |
| *3* | 4,635 (21) | 4,584 (21) |  |
| *2* | 4,345 (20) | 4,289 (20) |  |
| *1* | 3,621 (17) | 3,578 (16) |  |
| **Frailty Rating** |  |  | <0.001 |
| *Fit* | 13,853 (63) | 13,447 (62) |  |
| *Mild* | 6,864 (31) | 6,980 (32) |  |
| *Moderate* | 1,004 (4.6) | 1,282 (5.9) |  |
| *Severe* | 101 (0.5) | 113 (0.5) |  |
| **Smoking** |  |  | 0.30 |
| *Non-smoker* | 9,561 (44) | 9,668 (44) |  |
| *Smoker* | 12,261 (56) | 12,154 (56) |  |
| **Alcohol excess** |  |  | 0.041 |
| *Alcohol excess* | 438 (2.0) | 500 (2.3) |  |
| *No alcohol excess* | 21,384 (98) | 21,322 (98) |  |
| **Obesity** |  |  | <0.001 |
| *Not obese* | 18,539 (85) | 18,006 (83) |  |
| *Obese* | 3,283 (15) | 3,816 (17) |  |
| **Duration of follow-up (years)** | 11.92 (8.24 – 11.92) | 11.92 (11.82 – 11.92) | <0.001 |
| **start_year_strata** |  |  | <0.001 |
| *2010-2013* | 21,369 (98) | 21,240 (97) |  |
| *2014-2017* | 423 (1.9) | 535 (2.5) |  |
| *2018-2022* | 30 (0.1) | 47 (0.2) |  |
| **Outcome** |  |  | <0.001 |
| *Alive, no dementia* | 16,041 (74) | 18,300 (84) |  |
| *Developed dementia* | 1,636 (7.5) | 1,145 (5.2) |  |
| *Died* | 4,145 (19) | 2,377 (11) |  |
| ^1^Median (IQR); n (%) | | | |
| ^2^Wilcoxon rank sum test; Pearson's Chi-squared test; Fisher's exact test | | | |

## Table 3. Lung cancer propensity matched cohort demographics

|  | **Bloodstream infection** | |  |
| --- | --- | --- | --- |
| **Variable** | **No BSI**  N = 29,054^1^ | **BSI**  N = 29,054^1^ | **p-value**^2^ |
| **Age (years)** | 69 (57 – 78) | 68 (56 – 77) | <0.001 |
| **Sex** |  |  | 0.035 |
| *Male* | 15,310 (53) | 15,564 (54) |  |
| *Female* | 13,744 (47) | 13,490 (46) |  |
| **Charlson Index** |  |  | 0.005 |
| *Up to 20* | 28,779 (99) | 28,701 (99) |  |
| *>20* | 275 (0.9) | 353 (1.2) |  |
| **Welsh Index of Multiple Deprivation** |  |  | 0.91 |
| *5* | 5,142 (18) | 5,093 (18) |  |
| *4* | 5,289 (18) | 5,346 (18) |  |
| *3* | 5,680 (20) | 5,660 (19) |  |
| *2* | 6,425 (22) | 6,482 (22) |  |
| *1* | 6,518 (22) | 6,473 (22) |  |
| **Frailty Rating** |  |  | <0.001 |
| *Fit* | 15,162 (52) | 14,869 (51) |  |
| *Mild* | 10,073 (35) | 9,589 (33) |  |
| *Moderate* | 3,286 (11) | 3,788 (13) |  |
| *Severe* | 533 (1.8) | 808 (2.8) |  |
| **Smoking** |  |  | 0.20 |
| *Non-smoker* | 10,396 (36) | 10,544 (36) |  |
| *Smoker* | 18,658 (64) | 18,510 (64) |  |
| **Alcohol excess** |  |  | 0.027 |
| *Alcohol excess* | 1,664 (5.7) | 1,790 (6.2) |  |
| *No alcohol excess* | 27,390 (94) | 27,264 (94) |  |
| **Obesity** |  |  | <0.001 |
| *Not obese* | 25,256 (87) | 24,865 (86) |  |
| *Obese* | 3,798 (13) | 4,189 (14) |  |
| **BSI organism** |  |  | 0.56 |
| *E. coli* |  | 17,679 (61) |  |
| *Klebsiella* |  | 3,187 (11) |  |
| *MRSA* |  | 834 (2.9) |  |
| *MSSA* |  | 5,738 (20) |  |
| *Polymicrobial* |  | 574 (2.0) |  |
| *PsA* |  | 1,042 (3.6) |  |
| **Duration of follow-up (years)** | 10.7 (5.3 – 11.9) | 9.5 (5.7 – 11.9) | <0.001 |
| **start_year_strata** |  |  | <0.001 |
| *2010-2013* | 28,268 (97) | 28,051 (97) |  |
| *2014-2017* | 660 (2.3) | 820 (2.8) |  |
| *2018-2022* | 126 (0.4) | 183 (0.6) |  |
| **Outcome** |  |  | <0.001 |
| *Alive, no cancer* | 17,152 (59) | 12,436 (43) |  |
| *Lung cancer* | 647 (2.2) | 207 (0.7) |  |
| *Died* | 11,255 (39) | 16,411 (56) |  |
| ^1^Median (IQR); n (%) | | | |
| ^2^Wilcoxon rank sum test; Pearson's Chi-squared test; Fisher's exact test | | | |

## Table 4. Bloodstream infection cohort demographics split by Gram stain

|  | **Bloodstream infection** | |  |
| --- | --- | --- | --- |
| **Variable** | **Gram negative**  N = 18,959^1^ | **Gram positive**  N = 5,886^1^ | **p-value**^2^ |
| **Age (years)** | 74 (64 – 82) | 68 (54 – 79) | <0.001 |
| **Sex** |  |  | <0.001 |
| *Male* | 9,523 (50) | 3,770 (64) |  |
| *Female* | 9,436 (50) | 2,116 (36) |  |
| **Charlson Index** | 0 (0 – 0) | 0 (0 – 0) | 0.029 |
| **Welsh Index of Multiple Deprivation** |  |  | <0.001 |
| *5* | 3,413 (18) | 924 (16) |  |
| *4* | 3,524 (19) | 1,028 (17) |  |
| *3* | 3,702 (20) | 1,151 (20) |  |
| *2* | 4,151 (22) | 1,327 (23) |  |
| *1* | 4,169 (22) | 1,456 (25) |  |
| **Frailty Rating** |  |  | <0.001 |
| *Fit* | 9,742 (51) | 3,331 (57) |  |
| *Mild* | 6,262 (33) | 1,778 (30) |  |
| *Moderate* | 2,435 (13) | 643 (11) |  |
| *Severe* | 520 (2.7) | 134 (2.3) |  |
| **Charlson_strata_inf** |  |  | <0.001 |
| *Up to 20* | 15,347 (81) | 4,611 (79) |  |
| *21-40* | 3,350 (18) | 1,176 (20) |  |
| *41-50* | 174 (0.9) | 63 (1.1) |  |
| *>50* | 43 (0.2) | 23 (0.4) |  |
| **FRAILTY_FST_INF** |  |  | <0.001 |
| *Fit* | 5,259 (28) | 2,012 (34) |  |
| *Mild* | 7,102 (37) | 2,061 (35) |  |
| *Moderate* | 4,756 (25) | 1,315 (22) |  |
| *Severe* | 1,842 (9.7) | 498 (8.5) |  |
| **Smoking** |  |  | <0.001 |
| *Non-smoker* | 5,898 (31) | 1,645 (28) |  |
| *Smoker* | 13,061 (69) | 4,241 (72) |  |
| **Alcohol excess** |  |  | <0.001 |
| *Alcohol excess* | 1,602 (8.4) | 913 (16) |  |
| *No alcohol excess* | 17,357 (92) | 4,973 (84) |  |
| **Obesity** |  |  | 0.83 |
| *Not obese* | 15,117 (80) | 4,701 (80) |  |
| *Obese* | 3,842 (20) | 1,185 (20) |  |
| **Attribution** |  |  | <0.001 |
| *community* | 10,126 (53) | 2,603 (44) |  |
| *hospital* | 8,833 (47) | 3,282 (56) |  |
| *Missing* | 0 | 1 |  |
| **CRP_strata** |  |  | <0.001 |
| *Under 100* | 3,030 (18) | 997 (19) |  |
| *100-200* | 5,140 (30) | 1,387 (27) |  |
| *200-300* | 4,820 (28) | 1,347 (26) |  |
| *300-400* | 2,861 (17) | 960 (19) |  |
| *400+* | 1,157 (6.8) | 477 (9.2) |  |
| *Missing* | 1,951 | 718 |  |
| **Developed dementia** |  |  | <0.001 |
| *0* | 17,458 (92) | 5,622 (96) |  |
| *1* | 1,501 (7.9) | 264 (4.5) |  |
| **Outcome at 30 days** |  |  | <0.001 |
| *Alive* | 16,216 (86) | 4,628 (79) |  |
| *Died* | 2,743 (14) | 1,258 (21) |  |
| **Duration of follow-up (years)** | 1.61 (0.25 – 4.35) | 1.25 (0.11 – 4.33) | <0.001 |
| **Outcome** |  |  | <0.001 |
| *Alive, no dementia* | 8,009 (42) | 2,416 (41) |  |
| *Developed dementia* | 1,464 (7.7) | 254 (4.3) |  |
| *Died* | 9,486 (50) | 3,216 (55) |  |
| ^1^Median (IQR); n (%) | | | |
| ^2^Wilcoxon rank sum test; Pearson's Chi-squared test; Pearson's Chi-squared test with simulated p-value  (based on 2000 replicates) | | | |

## Table 5. Comparison of Gram-positive and Gram-negative BSI and risk of subsequent dementia

|  | **Unadjusted** | **Adjusted** |  |
| --- | --- | --- | --- |
| **Time (years)** | **Cumulative excess hazard**  **(95% CI)** | **Cumulative excess hazard**  **(95% CI)** | **Excess dementia**  **(per 1,000 patient years)** |
| 1 | -0.011 (-0.018 – -0.005) | 0.007 (0.000-0.014) | 6.9 (0.1 – 14) |
| 5 | -0.058 (-0.073 – -0.004) | 0.009 (0.006-0.025) | 9.3 (-6.0 – 25) |
| 10 | -0.094 (-0.124 – -0.064) | 0.022 (0.012-0.056) | 21.8 (-12 – 56) |

## Table 6. Bloodstream infection cohort demographics split by peak C-reactive protein

|  | **Bloodstream infection** | | | | |  |
| --- | --- | --- | --- | --- | --- | --- |
| **Variable** | **Under 100**  N = 4,100^1^ | **100-200**  N = 6,653^1^ | **200-300**  N = 6,292^1^ | **300-400**  N = 3,887^1^ | **400+**  N = 1,663^1^ | **p-value**^2^ |
| **Age (years)** | 74 (62 – 83) | 75 (65 – 84) | 73 (62 – 82) | 72 (60 – 80) | 68 (57 – 78) | <0.001 |
| **Sex** |  |  |  |  |  | <0.001 |
| *Male* | 2,303 (56) | 3,781 (57) | 3,346 (53) | 1,941 (50) | 706 (42) |  |
| *Female* | 1,797 (44) | 2,872 (43) | 2,946 (47) | 1,946 (50) | 957 (58) |  |
| **Charlson Index** | 0 (0 – 0) | 0 (0 – 0) | 0 (0 – 0) | 0 (0 – 0) | 0 (0 – 0) | 0.31 |
| **Welsh Index of Multiple Deprivation** |  |  |  |  |  | 0.10 |
| *5* | 668 (16) | 1,155 (17) | 1,044 (17) | 599 (15) | 279 (17) |  |
| *4* | 771 (19) | 1,241 (19) | 1,210 (19) | 683 (18) | 301 (18) |  |
| *3* | 832 (20) | 1,379 (21) | 1,225 (19) | 764 (20) | 337 (20) |  |
| *2* | 907 (22) | 1,463 (22) | 1,396 (22) | 930 (24) | 379 (23) |  |
| *1* | 922 (22) | 1,415 (21) | 1,417 (23) | 911 (23) | 367 (22) |  |
| **Frailty Rating** |  |  |  |  |  | <0.001 |
| *Fit* | 2,103 (51) | 3,446 (52) | 3,431 (55) | 2,201 (57) | 959 (58) |  |
| *Mild* | 1,356 (33) | 2,180 (33) | 1,983 (32) | 1,200 (31) | 513 (31) |  |
| *Moderate* | 525 (13) | 860 (13) | 732 (12) | 416 (11) | 150 (9.0) |  |
| *Severe* | 116 (2.8) | 167 (2.5) | 146 (2.3) | 70 (1.8) | 41 (2.5) |  |
| **Charlson_strata_inf** |  |  |  |  |  | <0.001 |
| *Up to 20* | 3,224 (79) | 5,250 (79) | 5,088 (81) | 3,189 (82) | 1,401 (84) |  |
| *21-40* | 802 (20) | 1,293 (19) | 1,128 (18) | 647 (17) | 241 (15) |  |
| *41-50* | 53 (1.3) | 75 (1.1) | 42 (0.7) | <40 | 16 (1.0) |  |
| *>50* | 14 (0.3) | 18 (0.3) | 19 (0.3) | <10 | 0 (0) |  |
| *Missing* | 7 | 17 | 15 | 12 | 5 |  |
| **FRAILTY_FST_INF** |  |  |  |  |  | <0.001 |
| *Fit* | 1,130 (28) | 1,770 (27) | 1,850 (29) | 1,211 (31) | 569 (34) |  |
| *Mild* | 1,527 (37) | 2,383 (36) | 2,332 (37) | 1,477 (38) | 637 (38) |  |
| *Moderate* | 1,022 (25) | 1,778 (27) | 1,522 (24) | 910 (23) | 343 (21) |  |
| *Severe* | 421 (10) | 722 (11) | 588 (9.3) | 289 (7.4) | 114 (6.9) |  |
| **Smoking** |  |  |  |  |  | 0.93 |
| *Non-smoker* | 1,232 (30) | 1,985 (30) | 1,880 (30) | 1,151 (30) | 512 (31) |  |
| *Smoker* | 2,868 (70) | 4,668 (70) | 4,412 (70) | 2,736 (70) | 1,151 (69) |  |
| **Alcohol excess** |  |  |  |  |  | <0.001 |
| *Alcohol excess* | 585 (14) | 672 (10) | 554 (8.8) | 324 (8.3) | 143 (8.6) |  |
| *No alcohol excess* | 3,515 (86) | 5,981 (90) | 5,738 (91) | 3,563 (92) | 1,520 (91) |  |
| **Obesity** |  |  |  |  |  | <0.001 |
| *Not obese* | 3,347 (82) | 5,354 (80) | 4,995 (79) | 3,013 (78) | 1,255 (75) |  |
| *Obese* | 753 (18) | 1,299 (20) | 1,297 (21) | 874 (22) | 408 (25) |  |
| **Attribution** |  |  |  |  |  | <0.001 |
| *community* | 1,957 (48) | 3,246 (49) | 3,352 (53) | 2,221 (57) | 1,001 (60) |  |
| *hospital* | 2,143 (52) | 3,407 (51) | 2,940 (47) | 1,666 (43) | 661 (40) |  |
| *Missing* | 0 | 0 | 0 | 0 | 1 |  |
| **ORG_SPEC** |  |  |  |  |  | <0.001 |
| *E. coli* | 2,443 (60) | 4,199 (63) | 3,934 (63) | 2,323 (60) | 929 (56) |  |
| *Klebsiella* | 464 (11) | 729 (11) | 699 (11) | 385 (9.9) | 158 (9.5) |  |
| *MRSA* | 104 (2.5) | 159 (2.4) | 156 (2.5) | 109 (2.8) | 40 (2.4) |  |
| *MSSA* | 893 (22) | 1,228 (18) | 1,191 (19) | 851 (22) | 437 (26) |  |
| *Polymicrobial* | 73 (1.8) | 126 (1.9) | 125 (2.0) | 66 (1.7) | 29 (1.7) |  |
| *PsA* | 123 (3.0) | 212 (3.2) | 187 (3.0) | 153 (3.9) | 70 (4.2) |  |
| **Outcome at 30 days** |  |  |  |  |  | <0.001 |
| *Alive* | 3,569 (87) | 5,732 (86) | 5,255 (84) | 3,118 (80) | 1,248 (75) |  |
| *Died* | 531 (13) | 921 (14) | 1,037 (16) | 769 (20) | 415 (25) |  |
| **Duration of follow-up (years)** | 1.45 (0.26 – 4.06) | 1.34 (0.25 – 3.71) | 1.44 (0.18 – 4.07) | 1.61 (0.15 – 4.42) | 1.37 (0.07 – 4.28) | <0.001 |
| **Outcome** |  |  |  |  |  | <0.001 |
| *Alive, no dementia* | 1,614 (39) | 2,738 (41) | 2,778 (44) | 1,807 (46) | 813 (49) |  |
| *Developed dementia* | 359 (8.8) | 522 (7.8) | 400 (6.4) | 176 (4.5) | 60 (3.6) |  |
| *Died* | 2,127 (52) | 3,393 (51) | 3,114 (49) | 1,904 (49) | 790 (48) |  |
| ^1^Median (IQR); n (%) | | | | | | |
| ^2^Kruskal-Wallis rank sum test; Pearson's Chi-squared test; Fisher's Exact Test for Count Data with simulated p-value  (based on 2000 replicates) | | | | | | |

## Table 7. Association between peak C-reactive protein (CRP) with subsequent dementia

|  | **Unadjusted** | **Adjusted** |  |
| --- | --- | --- | --- |
| **Time (years)** | **Cumulative excess hazard**  **(95% CI)** | **Cumulative excess hazard**  **(95% CI)** | **Excess dementia**  **(per 1,000 patient years, per 50mg/L increase in peak CRP)** |
| 1 | -0.005 (-0.005 to -0.004) | -0.003 (-0.003 to -0.002) | -2.5 (-2.7 to -2.3) |
| 5 | -0.014 (-0.015 to -0.014) | -0.008 (-0.008 to -0.008) | -8.1 (-8.5 to -7.7) |
| 10 | -0.019 (-0.020 to -0.017) | -0.010 (-0.011 to -0.009) | -10.2 (-11.2 to -9.1) |
